# G-quadruplexes formation in the 5’UTRs of mRNAs associated with colorectal cancer pathways

**DOI:** 10.1371/journal.pone.0208363

**Published:** 2018-12-03

**Authors:** Rachel Jodoin, Jean-Pierre Perreault

**Affiliations:** Département de Biochimie, Faculté de médecine et des sciences de la santé, Université de Sherbrooke, Sherbrooke, Québec, Canada; University of Surrey, UNITED KINGDOM

## Abstract

RNA G-quadruplexes (rG4) are stable non-canonical secondary structures composed of G-rich sequences. Many rG4 structures located in the 5’UTRs of mRNAs act as translation repressors due to their high stability which is thought to impede ribosomal scanning. That said, it is not known if these are mRNA-specific examples, or if they are indicative of a global expression regulation mechanism of the mRNAs involved in a common pathway based on structure folding recognition. Gene-ontology analysis of mRNAs bearing a predicted rG4 motif in their 5’UTRs revealed an enrichment for mRNAs associated with the colorectal cancer pathway. Bioinformatic tools for rG4 prediction, and experimental *in vitro* validations were used to confirm and compare the folding of the predicted rG4s of the mRNAs associated with dysregulated pathways in colorectal cancer. The rG4 folding was confirmed for the first time for 9 mRNAs. A repressive effect of 3 rG4 candidates on the expression of a reporter gene was also measured in colorectal cancer cell lines. This work highlights the fact that rG4 prediction is not yet accurate, and that experimental characterization is still essential in order to identify the precise rG4 folding sequences and the possible common features shared between the rG4 overrepresented in important biological pathways.

## Introduction

RNA G-quadruplexes (rG4) are non-canonical secondary structures based on the stacking of multiple g-quartets. A G-quartet is a coplanar array of four guanines (G) linked by Hoogsteen base-pairs and stabilized in its center by a monovalent cation, usually K^+^. In the recent years, many rG4 located in the 5’UTRs of mRNAs have been described [1–3]. To date, at least 35 examples of rG4 folding affecting expression levels *in cellulo* have been reported, including that of the well-known oncogene N-Ras [4]. Based on a search for canonical motifs only, more than 2 000 human 5’UTRs were predicted to possess potential rG4 structures (PG4) [5,6]. Experimentally, a recent study using a next-generation sequencing technique called rG4-seq showed an enrichment of rG4 in the UTRs of mRNAs [7]. More specifically in the 5’UTR, 540 regions appeared to fold into the structure, many of these with sequences features that were divergent from the canonical description of an rG4 motif.

There is evidence of rG4 formation in the cell cytoplasm from experiments using both fluorescent antibodies and chemical probes specific for the structure [8,9]. However, recent work using *in vivo* DMS-probing demonstrated that, in eukaryotes, rG4 are mostly unfolded [10]. This could indicate that the rG4 motifs identified in the transcriptome are either prevented from folding, are actively unfolded, or that the rG4 structures might be transient and folded only in specific regulatory mechanisms. Actually, some RNA-binding proteins are known to specifically recognize and bind rG4 structures (reviewed in [2]). Helicases such as DHX36 [11], DDX21 [12] and DHX9 [13] unfold the structure. Due to the high stability of the structure, rG4s in 5’UTRs were defined primarily as translational repressors that impaired ribosomal scanning [14]. There are also a few examples of 5’UTR rG4s acting as translational enhancers, or as a part of the internal ribosome entry sites that are important for cap-independent translation [15–17].

At the DNA level, G4 probable sequences are enriched in oncogenes and depleted in tumor suppressor genes [18]. Recent work also demonstrated that G4 located in the promoters of DNA repair genes are folded and might play a role in the oxidative stress response [19]. At the RNA level, Kwok *et al*. observed an enrichment of rG4-seq detected rG4 in the mRNAs of genes involved in RNA processing, stability and transcription regulation [7]. Other than translation, mRNA-specific rG4s were also studied for their roles in post-transcriptional processes [2,20], including some that are important in diseases such as cancer or neuropathology [3]. Actually, G4 structures are considered as potential therapeutic targets and multiple efforts are driven toward the rational design of small-molecule ligands that would target the rG4 structure in specific mRNA transcripts [21]. All of this transcript-specific evidence points to the hypothesis that, similar to the DNA G4s located in promoters, rG4s located in the 5’UTRs might be structural motifs responsible for the co-regulation of the expression levels of mRNAs with different functions in order to regulate either global pathways or cellular responses.

The primary method for identifying potential G4 (PG4) is the presence of the consensus sequence motif. The canonical G4 were originally described as G_x_-N_1-7_-G_x_-N_1-7_-G_x_-N_1-7_-G_x_, where x ≥ 3 [5]. The consecutive Gs form the four essential G-tracts that are linked by three series of any of the four nucleotides (N) that are called loops. However, extensive studies have now characterized rG4 folding for a broader array of motifs: the stacking of only two quartets [22], or of more than three [23], the presence of loops longer than seven nucleotides [24,25], the presence of bulges in the G-tracts [26], or even completely different and unpredictable motifs such as the G-quadruplexes of the fluorescent RNA aptamers Spinach and Mango [27,28]. All of which renders the prediction of rG4 formation extremely difficult with a large spectrum of possible motifs. Moreover, the canonical motif in itself is not enough to result in rG4 formation, as the nucleotide context of the motif, for example the presence of C-rich sequences, can compete with rG4 formation and favor the formation of a double-stranded RNA structure (dsRNA) instead. This observation lead to the development of several G4 prediction tools that can measure G4 propensity, including measuring the competing nucleotide context (cG/cC score and G4H) [29,30], the G4 homology by comparing to experimentally confirmed rG4 (G4NN) [31] and predicting the possible secondary structures in order to identify the most stable one (RNAfold) [32].

Many biophysical techniques exist that can be used to both confirm the presence of and characterize rG4 folding, techniques such as a specific CD spectra signature, UV-absorbance and thermal denaturation [33]. Dyes also provide fluorescence enhancement upon binding to specific G4 topologies [34,35]. All of these techniques offer a global idea of the folding as either dsRNA or rG4, but none precisely define the nucleotides involved in either the base-pairs or the G-tracts of the rG4. In addition, these techniques can be used only with short nucleotide sequences. *In-line* probing is specific for RNA sequences and it was adapted to rG4 probing [36]. Compared to the above mentioned methods for studying the rG4 motif, it can be performed with longer sequences, which makes it useful in the competing nucleotide context, and it generates significantly more information about the flexibility of the individual residues in the structure.

As potential rG4 structures in 5’UTRs are pervasive and biologically relevant, whether or not the presence of rG4 in the 5’UTRs of different mRNAs could be related to their common regulation, or association with a similar pathway, were investigated using gene-ontology enrichment. As rG4 are difficult to predict based solely on the presence of the motif, the accessible tools for the prediction of rG4 were used and their results were compared to those of experiments using *in-line* probing and fluorescence enhancement assays that confirmed the folding *in vitro*. This permitted the observation of the rG4s features that are shared by the mRNAs of the same pathway ontology, as well as the *in cellulo* measurement of the rG4s effects of some candidates using gene-reporter expression assays. This work sheds light on both the remaining flaws of the rG4 prediction tools, and on the importance of the experimental characterization of individual rG4 in order to accurately identify them as they are possible mRNAs structural co-regulatory motifs.

## Material and methods

### PG4 database

Databases of PG4 located in the 5’UTRs of mRNAs corresponding to the canonical motif G_3_-N_1-7_-G_3_-N_1-7_-G_3_-N_1-7_-G_3_ are available from Beaudoin and Perreault [6] and PG4 with a longer central loop G_3_-N_1_-G_3_-N_1-20_-G_3_-N_1_-G_3_ from Jodoin *et al*. [24]. Briefly, the databases were built by retrieving all 5’UTR sequences from the database UTRdb [37]. Python scripts were then used to search for the sequence motif and to identify its position.

### Bioinformatic methods

The gene ontology enrichment analyses were performed using the DAVID bioinformatic resources version 6.7. A list of the 2 004 mRNAs’ 5’UTRs containing at least 1 PG4 corresponding to either the canonical sequence or one with a central loop up to 20 nts in length were compared to the background of all human 5’UTR mRNAs (a total number of 31 654). Pathways were recovered using the KEGG orthology database [38]. All of the candidates’ mRNAs information (RefSeq number, UTRdb ID and Kegg orthology or AmiGO annotations [39,40]) are reported in S3 Table.

Following the selection of the 26 PG4 candidates, the cG/cC [29], the G4Hunter [30] and the G4NN scores [31] were measured using the G4 screener webserver [41]. The thresholds selected for rG4 formation were 3.0, 0.9 and 0.5, respectively, so as to maximize both sensitivity and selectivity. For the RNA sequences used in the *in vitro* experiments, the scoring window was set to 200 nts in order to include the full-lengths of all sequences, and to give only one value for each score.

RNA secondary structure prediction of the sequences used for the *in vitro* experiments was performed using the RNAfold tool version 2.1.0 from the VIENNA RNA suite [42] and changing the default parameters in order to add g-quadruplex predictions. The resulting most stable secondary-structures are represented by dot-bracket notation, and the guanines predicted to fold into rG4 are represented by the “+” symbol.

### Construction of RNA sequences

The sequences tested *in vitro* consist of the PG4 in question surrounded by 15 to 50 nts of its natural 5’ and 3’ contexts. The G/A-mutants were designed to disrupt the G-tracts. Hence, each second G of a tract was mutated to A (typical examples: GGG were mutated to GAG, GGGG to GAGA or GAAG, etc.). The 17 nts T7 promoter sequence (TAATACGACTCACTATA) were added for *in vitro* transcription purposes, followed by 2 or 3 Gs if they were not already present in the 5’UTR. The sequences of all of the oligonucleotides used are presented in S4 Table. PCR templates for *in vitro* transcription were obtained by PCR filling of the 2 complementary oligonucleotides (IDT or Invitrogen) using purified PFU DNA polymerase (12 cycles of 1 min each at 95°C, 54°C and 72°C, followed by a final elongation at 72°C for 5 min) in buffer containing 0.2 mM dNTPs, 2 mM MgSO_4_, 20 mM Tris-HCl pH 8.8, 10 mM KCl, 10 mM (NH_4_)_2_SO_4_, 0.1% Triton-X-100 and 5 mM DMSO. The DNA template sizes were verified by agarose gel electrophoresis. The PG4 DNA templates were ethanol precipitated, dried and dissolved in 50 μL H_2_O. *In vitro* T7 RNA transcription reactions were performed for 2 h at 37°C using 10 μg of purified T7 polymerase in a solution with 5 mM rNTPs, 0.01 U pyrophosphatase (Roche Diagnostics), 80 mM HEPES-KOH pH 7.5, 24 mM MgCl_2_, 40 mM DTT and 2 mM spermidine. In order to remove the DNA template and to remove protein contaminants from the transcription reactions, a DNAse treatment (RQ1 DNAse, Promega) followed by phenol-chloroform extraction and ethanol precipitation were performed. The recovered RNA was separated on an 8% denaturing polyacrylamide gel (PAGE; 19:1 ratio acrylamide to bisacrylamide, 8 M urea using 45 mM Tris-borate pH 7.5 and 1 mM EDTA solution as running buffer). The bands were visualized by UV-shadowing, the corresponding gel slices were cut out and the RNA eluted in elution buffer (1 mM EDTA, 0.1% SDS and 0.5 M ammonium acetate) and ethanol precipitated. RNA was dissolved in water and quantified using a Nanodrop Lite spectrophotometer (ThermoFisher scientific).

#### 5’ end-labelling of RNA transcript

RNA (50 pmol) was dephosphorylated in a 50 μL reaction using Antarctic phosphatase (1 U, New England Biolabs) using the manufacturer’s protocol. The enzyme was inactivated by heating to 65°C for 7 min. Then, 10 pmol of dephosphorylated RNA was kept and γ^32^P-ATP (2 μL; 6 000 Ci (222 TBq)/mmol in 50 mM Tricine pH 7.6, PerkinElmer) was added along with 3 U of T4 Kinase (Promega) and the reaction was incubated for 1 h at 37°C. Labeled RNA was separated by denaturing PAGE as described previously. The RNA was detected by autoradiography. The correct RNA band was cut out of the gel and the RNA eluted, ethanol precipitated and dissolved in 30 μL of water. The radioactivity in counts-per-minute (cpm) for each sample was measured using a single-well gamma particle counter (Bioscan QC-2000).

### *In-line* probing

*In-line* probing was performed as described previously [36]. Briefly, the probing of each candidate was performed in duplicate from two different *in vitro* transcription reactions. Equal amounts, in terms of cpm (50 000 cpm), of 5’end labeled WT and G/A-mutant sequences were dissolved in folding buffer (10 μL; 20 mM Li cacodylate pH 7.5 and 100 mM of either LiCl or KCl). Folding was performed by heating the RNA sample for 5 min à 70°C followed by a 1 h slow-cool to room temperature (RT). *In-Line* 2X buffer (50 μL, 40 mM Li cacodylate pH 8.5, 40 mM MgCl_2_ and 200 mM of either LiCl or KCl) and 40 μL H_2_O were then added so as to obtain a final volume of 100 μL. The samples were incubated for 40 h at RT in order to allow for self-cleavage to occur. The RNA was then ethanol-precipitated and dissolved in 20 μL of denaturing loading buffer (95% formamide, 10 mM EDTA, 0.025% xylene cyanol). Before separation on a 10% denaturing PAGE, the cpm of each sample was measured and the sample diluted if necessary so as to load an equal cpm amount of each. Both alkaline hydrolysis and RNase T1 sequence ladders where migrated alongside the samples. In order to obtain the alkaline hydrolysis ladder, a 5 μL solution of 50 000 cpm of either WT or G/A-mutant 5’end labeled RNA was treated with 2 μL of 2 N NaOH for 1 min at RT. The reaction was stopped by the addition of 3 μL of 1 M Tris-HCl pH 7.5. The RNA was then ethanol-precipitated and dissolved in 20 μl of denaturing loading buffer. In order to obtain the RNase T1 ladder, an 8 μL solution of 50 000 cpm of either WT or G/A-mutant 5’end labeled RNA was treated for 2 min at 37°C with 1 μL of RNAse T1 enzyme (0.6 U, Roche Diagnostic) and 1 μL of 10X buffer (200 mM Tris-HCL pH 7.5, 100 mM MgCl_2_, 1 M LiCl). The reactions were stopped by the addition of 20 μL of denaturing loading buffer. The gel was migrated for 2 h at 60 Watts. The gel was then put on a Whatman paper and dried for 45 min at 80°C in a gel drier. The dried gel was exposed to a phosphorimager screen overnight, and cleavage pattern visualized using a Typhoon Trio imaging system (GE Healthcare).

#### Quantification of the *in-line* probing data

Quantification of gel band intensities was performed using the SAFA semi-automated software [43]. Equal loading in the K^+^ and Li^+^ lanes of the *in-line* probing gels was first verified by quantifying the intensity of the top unresolved band. If there was less than a 15% difference the loading was considered as being equal, and the ratios of the intensities could be calculated. The intensities of each band in the K^+^ conditions were divided by the intensities of each related band in the Li^+^ conditions. The average K^+^/Li^+^ ratios of two independent experiments were represented on a graph for each nucleotide and for each condition (WT or G/A-mutant). The error bars represent the standard deviation. A K^+^/Li^+^ ratio threshold of 2 was set so as to conclude that a difference in nucleotide flexibility existed in the K^+^ condition.

### NMM fluorescence assay

The RNA WT and G/A-mutant sequences used for the NMM fluorescence assay were the same as those used for the *in-line* probing experiments. After *in vitro* transcription, the RNA was quantified using a Nanodrop Lite spectrophotometer (ThermoFisher scientific). RNA (200 pmol) was dissolved in 50 μL of the same folding buffer as was used in the *in-line* probing experiments. The RNA was heated to 70°C for 5 min, and then was slow-cooled to RT for 1 h. *In-line* 2X buffer (50 μL) was then added. NMM 0.5 mM (1 μL; N-Methyl-Mesoporphyrin IX, NMM580, Frontier Scientific Inc., Logan, Utah,) was then added and the mix was incubated for 30 min at RT in the dark. Fluorescence spectrophotometry was performed using a Hitachi F-2500 fluorescence spectrophotometer with an excitation bandwidth of 399 nM, and the emission spectra was measured from 550 to 650 nm in a 10 mm quartz cuvette. The fluorescence units of the maximal peak at 605 nm was used to compare the Li^+^ and K^+^ conditions. The experiments were performed in triplicate with RNA sequences from three independent *in vitro* transcription reactions. The results are presented as the differences of the means of the K^+^ and Li^+^ fluorescence peaks at 605 nm.

### Cell cultures

HEK293 cells (origin ATCC, CRL-1573) were cultivated in Dulbecco’s modified Eagle medium (DMEM) supplemented with 10% foetal bovine serum (FBS). HCT116 and HT-29 cells (origin ATCC, CCL-247 and ATCC, HTB-38, respectively) were cultivated in McCoy’s 5A medium supplemented with 10% FBS. DLD-1 cells (origin ATCC, CCL-221) were cultivated in Roswell Park Memorial Institute 1640 medium (RPMI) supplemented with 10% FBS. In every case the incubations were performed in an incubator at 37°C with a 100% H_2_O and 5% CO_2_ atmosphere. All cell culture reagents were purchased from Multicell and Wisent.

### Cloning and transfection

Full length WT 5’UTR and G/A-mutant sequence constructs (the same mutations as were used in the *in vitro* experiment) with *NheI* restriction sites at both ends were generated by PCR filling of two complementary DNA oligonucleotides (Invitrogen) using the same protocol as described in the section: Construction of RNA sequences. The sequences and primers used for cloning are listed in the S5 Table. Full length 5’UTR constructs with the *NheI* restriction sites were digested and ligated into the *Renilla* luciferase (Rluc) pRL-TK vector (Promega). The correct insertions and mutations were verified by DNA sequencing. The Firefly luciferase (Fluc) PGL3 vector (Promega) was used as a transfection control.

Twenty-four hours prior to transfection, HEK293 cells were seeded at 130 000 cells/well, while HCT116, HT-29 and DLD-1 cells were seeded at 190 000 cells/well in a 24-wells plates. Plasmid DNA (500 ng in total, 450 ng of the pRL-TK construction and 50 ng of pGL3 as control) were co-transfected using 0.5 μL/well of Lipofectamine 2000 as recommended by the manufacturer (Invitrogen) in the appropriate serum-free media for each cell type. Each candidate’s WT and G/A-mutant constructions were transfected in triplicate, and each experiment was repeated at least twice for each candidate.

### Dual-luciferase assays

Dual-luciferase assays were performed at RT using the manufacturer’s protocol (Dual-luciferase reporter assay system, Promega). Briefly, cell lysis was performed 24 h post-transfection with 150 μL (HEK293) or 100 μL (HCT116, HT-29 and DLD-1) of passive lysis buffer 5X. A volume of 3 to 10 μl of cell lysate was used for the assay. The luciferase reagents (100 μL each) were added sequentially. Luminescence readings were performed with a Glomax 20/20 luminometer. The read integration times was 10 sec. The results are presented as the means and standard deviations of the Rluc/Fluc ratios of at least two independent experiments.

### Statistical analyses

Statistical analyses were performed using GraphPad Prism 7.03 [44].

## Results and discussion

### Potential 5’UTR rG4 folding motifs are enriched in annotated pathways

Numerous rG4 structures located in the 5’UTRs of individual mRNAs have been identified as translational repressors. However, it is not known if rG4 motifs are enriched in particular mRNA families or in certain cellular pathways. In order to answer this question, a gene ontology-enrichment (GO-enrichment) analysis was performed using the DAVID bioinformatic tool [45]. The previously described database of potential rG4 (PG4) developed by Beaudoin and Perreault was used [6]. This database contains all of the 5’UTR sequences extracted from the UTRdb database [37] that contain the canonical motif G_3_-N_1-7_-G_3_-N_1-7_-G_3_-N_1-7_-G_3_. Based on previous findings from our group indicating that rG4 with longer loops can fold with good stability, and can also affect gene expression [24], the original database was conservatively extended to include 5’UTRs containing the motif G_3_-N_1_-G_3_-N_1-20_-G_3_-N_1_-G_3_. This represents PG4s with a central loop of 1 to 20 nucleotides (nts), while the two other loops are limited to one nucleotide each. Further extension of the sequence motif search was avoided so as to limit the number of false rG4 predictions. The final list of 2 004 PG4 mRNAs was then compared to the background of all *homo sapiens* GO-annotated mRNAs in search of possible enrichment in certain biological pathways. The GO-annotations related to pathways were taken from the KEGG pathways database [38]. The GO-annotations describe the molecular functions, the cellular components and the biological processes to which a gene and its corresponding mRNA transcripts are associated. Pathways annotations describe genes with various functions and cellular localizations that are commonly involved in higher order biological processes such as metabolic routes or the development of a disease. In the analysis performed here, an enrichment represents a higher proportion of the mRNAs in the PG4 mRNAs list being associated to a particular pathway as compared to the proportion of all mRNAs from the background that are associated with this same pathway. The results of the GO-enrichment analysis are presented in Table 1. Seven pathways presented an enrichment of 1.8- to 2.5-fold for mRNAs with a PG4 motif located in the 5’UTR with significant p-values. Three of these were cancer-related pathways: acute myeloid leukemia, chronic myeloid leukemia and colorectal cancer. The signaling pathways of neurotrophin and insulin were enriched, as was glycerophospholipid metabolism and endocytosis. Interestingly, many PG4 containing mRNAs were in common between the different pathways such as BAD, MAP2K1 and PIK3R1, which were present in five of them. These mRNAs code for proteins involved in signalization, stress response and proliferation; molecular functions that are commonly altered in diverse cancers [46], explaining why they are retrieved in multiple cancer related pathways.

**Table 1:** Gene ontology enrichment analysis.

Term	Count	%	P-Value	Genes	Fold Enrichment
hsa05221:Acute myeloid leukemia	11	0.87	0.001	CEBPA, NRAS, MAP2K1, STAT5A, MAPK3, STAT5B, RARA, BAD, PIK3R3, TCF7L1, PIK3R1	2.54
hsa00564:Glycerophospholipid metabolism	13	1.03	0.004	GPD2, CPT1B, DGKQ, LYPLA1, CDS1, CDS2, DGKZ, ETNK2, PCYT1B, PPAP2A, AGPAT2, CHAT, AGPAT1	2.54
hsa05220:Chronic myeloid leukemia	13	1.03	0.008	CTBP2, MAP2K1, STAT5A, STAT5B, SMAD4, BAD, ACVR1C, NRAS, CBLB, MAPK3, PIK3R3, CRK, PIK3R1	2.33
hsa05210:Colorectal cancer	14	1.11	0.010	MAP2K1, SMAD4, SMAD2, FZD2, BAD, APPL1, TCF7L1, ACVR1C, FZD10, CASP9, BCL2, MAPK3, PIK3R3, PIK3R1	2.18
hsa04722:Neurotrophin signaling pathway	20	1.59	0.002	YWHAZ, IRS2, MAP2K1, MAPK11, MAPKAPK2, BAD, NRAS, ATF4, PSEN1, PRDM4, MAP3K3, MAPK14, BCL2, MAPK3, SH2B3, NGFRAP1, SH2B1, PIK3R3, CRK, PIK3R1	2.13
hsa04910:Insulin signaling pathway	21	1.67	0.002	IRS2, MAP2K1, EXOC7, SOCS3, FLOT1, RHOQ, BAD, PPP1CC, PPP1CB, PRKAR2B, NRAS, PPP1CA, CBLB, PDPK1, INPP5K, MAPK3, PRKACA, PIK3R3, TRIP10, CRK, PIK3R1	2.05
hsa04144:Endocytosis	25	1.99	0.005	FGFR3, CHMP4B, CHMP6, ADRBK2, ARF6, ACVR1C, HSPA1L, RNF3, HSPA6, NEDD4L, IQSEC2, GIT1, PARD6A, EPN3, VPS45, RAB11FIP4, RAB11FIP5, CBLB, PSD, AP2A1, ARRB1, ACAP2, SMURF1, PARD6G, PIP4K2B	1.80

The biological significance of the GO-enrichment depends on the accurate prediction of rG4 formation in the mRNAs. It is probable that some PG4 of the initial 2 004 mRNAs sequences used for the GO-enrichment analysis are false positive predictions. However, this list represented the best starting point with which to consider pathway enrichment for the global amount of 5’UTR PG4s. Refinement of the rG4 prediction using different tools, and folding evaluation of the PG4 sequences of the selected enriched pathways, were the next steps towards the validation of the initial results.

### Dysregulated colorectal cancer pathways include mRNAs with PG4s

The colorectal cancer pathway was selected for the continuation of the investigation of the importance of rG4 motifs in mRNA expression regulation because of the important biological incidence of this cancer, and because the molecular aspects of its dysregulated pathways are well-characterized. Furthermore, of the 14 mRNAs containing PG4 located in the 5’UTR that are enriched in this pathway, 6 had been previously studied for rG4 formation and 4 were already known to adopt an rG4 conformation. These latter 4 were BCL-2 [47], FZD2 [6], ACVR1C and MAPK3 [29]. These results provided confidence that the GO-enrichment observed in this pathway was not biased by the presence of a high number of mRNAs with false rG4 predictions.

However, the KEGG’s list of the mRNAs associated with the colorectal cancer pathway does not include all of the mRNAs that are known to be dysregulated in this cancer type. In order to correct for the incomplete annotation, and to increase the number of candidates, the list was extended. To do so, the initial list of 2 004 mRNAs positive for the presence of PG4 motifs was re-analysed. The previous study of hundreds of colorectal cancer tumors analysed by the Cancer Genome Atlas Consortium defined four predominant dysregulated pathways: i.e. the WNT, TGF-β and PI3-Kinase signalling pathways, and the proliferation and apoptosis defects [48]. Twelve mRNAs from the PG4 database were thus recovered based on their more specific GO-annotations, related to one or more of the four colorectal cancer dysregulated pathways mentioned above (the GO-annotations of all candidates are listed in S3 Table).

Furthermore, one candidate that was not present in the initial PG4 mRNA list for the GO‑analysis, because it differed from the 1 to 20 nts central loop pattern, was also added. The APC candidate has a predicted central loop of 30 nts. The formation of an rG4 by this candidate had been previously confirmed by *in-line* probing [24]. Well-known to be mutated and important in colorectal cancer tumorigenesis [49], APC was added as a positive control for both rG4 formation and possession of a role in colorectal cancer. Table 2 presents the list of the 26 5’UTR PG4 candidates selected for further prediction and evaluation, regrouped by their associated mRNAs’ pathways.

**Table 2:** List of PG4 located in the 5’UTRs of mRNAs associated with colorectal cancer, their prediction of rG4 formation and their probing results.

Pathways	Candidates	rG4 predictions^1^				In vitro probing		rG4 formation^4^
		cG/cC	G4H	G4NN	RNA fold	*In-line* probing^2^	NMM fluorescence^3^	
WNT	APC	6.21	1.00	0.86	dsRNA	Yes	32.2	+
	BCL-9L	4.37	0.86	0.97	rG4	Yes	63.3	+
	FZD10	5.04	1.21	0.84	rG4	Yes	61.7	+
	FZD2	13.3	1.63	0.97	rG4	Yes	67.5	+
	TCF7L1	1.21	0.23	0.05	rG4	No	4.30	-
Apoptosis	AIFM2	4.33	0.89	0.35	rG4	Yes	49.3	+
	APPL1	1.92	0.46	0.20	dsRNA	Yes	46.1	+
	BAD	2.51	0.60	0.24	dsRNA	Yes	26.8	+
	BAG-1	4.04	0.83	0.62	rG4	Yes	82.0	+
	BAG-5	2.28	0.60	0.35	dsRNA	No	31.4	-
	BCL-2	1.93	0.44	0.05	dsRNA	Yes	52.9	+
	BOK	2.43	0.67	0.39	rG4	No	14.1	-
	CASP6	3.07	0.75	0.34	dsRNA	No	11.9	-
	CASP8AP2	3.57	0.79	0.77	rG4	Yes	70.8	+
	CASP9	3.68	1.06	0.63	rG4	No	51.9	-
TGF-β	ACVR1C	3.01	0.61	0.51	dsRNA	Yes	71.2	+
	BMPR1A	7.00	1.12	0.89	rG4	Yes	87.6	+
	SMAD2	1.59	0.29	0.03	dsRNA	No	35.2	-
	SMAD4 #1	2.54	0.70	0.31	rG4	No	10.8	-
	SMAD4 #2	3.07	0.82	0.47	dsRNA	No	12.2	-
	SMAD7	0.73	-0.05	0.02	dsRNA	No	0.30	-
	SMURF1	1.86	0.41	0.21	rG4	No	3.80	-
PI3-Kinase	MAP2K1	2.33	0.49	0.16	rG4	Yes	81.1	+
	MAPK3	9.00	1.63	0.97	rG4	Yes	48.1	+
	PIK3R1	2.97	0.83	0.43	rG4	Yes	35.6	+
	PIK3R3	2.13	0.52	0.21	rG4	No	5.10	-

^1.^ Thresholds for a positive rG4 prediction are ≥3.0 for cG/cC; ≥0.9 for G4H; and, ≥0.5 for G4NN

^2.^ Based on the cleavage pattern, “Yes” represents sequences with a K^+^/Li^+^ ratio of cleavage equal to or superior to the threshold of 2 for the nucleotides located between tracts of guanines that is characteristic of rG4 folding. “No” represents either a K^+^/Li^+^ ratio inferior to the threshold, or a higher ratio that is either inconsistent or insufficient for rG4 folding.

^3.^ Difference of the K^+^ and Li^+^ fluorescence emission peaks at 605 nm for the WT sequence.

^4.^ Assignment of rG4 formation, “+” represents sequence positive for rG4 folding, “-” represents sequence negative for rG4 folding based on the two *in vitro* probing assays.

### *In silico* predictions of rG4 formation vary between different tools

Previous work on rG4 have shown that their prediction based only on the presence of the sequence motif is prone to yielding many false positives [6,29,30]. Many factors can influence rG4 folding. For example, the presence of multiple cytosines (C) in the vicinity of the potential rG4 can compete with G-tract formation, resulting in G-C base pairs and folding into dsRNA instead of rG4. Therefore, a more detailed prediction of rG4 formation using the available bioinformatic tools was performed. The cG/cC and G4H scores were developed for RNA and DNA sequences, respectively [29,30]. They both use a similar window screening of sequences, but a different calculation method, to account for the possible unfavorable C-rich nucleotide context surrounding the potential rG4 motif. A third tool, the G4NN score [31], was recently developed to measure sequence homology to experimentally-confirmed positive and negative rG4 sequences from the G4RNA database [50]. Based on the sensitivity and specificity analyses of these previous studies which compared multiple PG4 sequences, the optimized thresholds for G4 prediction were set to 3.0, 0.9 and 0.5 for the cG/cC, G4H and G4NN scores, respectively. Finally, Lorenz *et al*. [32] developed an energy model for rG4 folding that was included in the RNAfold algorithm and allowed the comparison of the minimum free energies of the ensemble of dsRNA secondary structures versus that of the rG4 structure in order to identify the most stable one. Dot-and-bracket notation of the free or base-paired nucleotides of the secondary structure prediction was modified in order to add another symbol (+) indicating which Gs are involved in the G-tracts of the rG4. It was thus also used for the rG4 prediction for the set of 26 candidates.

The sequences selected for both the scoring and the secondary structure predictions were the PG4 sequence motifs obtained from the initial database. The sequence motifs ranged from 17 to 56 nts (3 overlapping PG4s) in size to obtain an average length of 27 nts. To account for the possible competitive secondary structure, a surrounding nucleotide context on either side (5’ and 3’) from the original 5’UTR was added. Based on our previous works [6,29], a nucleotide context ranging from 15 to 50 nts was ideal. The size of the added context for each of the 26 PG4 sequence depended on multiple factors. First, by the length of the 5’UTR, when possible the full 5’UTR was selected. Second, the size of the context was affected by the position of the PG4 motif inside the 5’UTR. The context was shorter if the PG4 was close to the 5’or 3’extremity of the UTR. The sequence context from the coding region of the transcript was never included. Third, in order to perform further *in vitro* folding evaluations, constraints on the maximal sequence length were considered. *In-line* probing can be performed on RNA sequences up to 150 nts long. Furthermore, RNA synthesis using T7 polymerase-driven *in vitro* transcription is favored by the presence of multiple guanines at the 5’ extremity, so the context size was selected to include natural 5’UTR context starting with multiple Gs when possible. Otherwise, 1 to 3Gs were added at the 5’-extremity. Considering all these technical limitations and each 5’UTR specificities, the resulting nucleotide context was of 4 to 60 nts in length, (average of 33 nts), and the resulting PG4 sequences studied were of 50 to 149 nts in length, with an average of 93 nts. The selected 26 PG4s sequences are available in S1 Table, and the predictions of their formation by the four tools are presented in Table 2. The scores were calculated using one window covering the entire sequence.

Overall, the predictions vary significantly from one tool to the next. The four predictors gave identical predictions of rG4 formation, or dsRNA formation, for only approximately one-half (i.e. 12) of the candidates. G4H has the lowest rG4 prediction numbers, with 6, and RNAfold has the highest with 16 predicted positive candidates out of the total of 26 (S2 Table). These divergent predictions result from the different stringencies of the tools in question.

### *In vitro* confirmation of rG4 folding

The rG4 predictors are a good starting point with which to identify strong PG4 candidates, but their accuracy can only be determined following experimental validation of the rG4 folding. To permit this evaluation, *in-line* probing cartography was performed on the same PG4 sequences and context used for prediction. The theoretical basis of this cartography is the self-cleaving potential of an RNA strand. The self-cleavage occurs when the flexible regions of the RNA strand adopt an “*in-line* conformation” between the 2’-hydroxyl group of the nucleotide’s ribose moiety and the phosphate group of the backbone. The secondary structure is inferred from both the cleaved pattern of the flexible nucleotides and the protected pattern of the base-paired nucleotides. This technique presents multiple advantages for rG4 probing that have been described previously [36]. For the 26 colorectal PG4 candidates, the WT sequence was compared with its corresponding G/A-mutant sequence in which at least one G of each predicted G-tract was mutated to adenine (A) in order to abolish all folding of the potential rG4. A second negative control was performed using lithium (Li^+^) instead of potassium (K^+^) in the buffer. K^+^ is essential for the stabilization of the G-quartets. The use of Li^+^ offers a similar ionic strength in solution, that is unfavorable for rG4 stabilization, but do not affect any other dsRNA base-pairing formation. During incubation, self-cleavage of the RNA occurs in the flexible nucleotide regions of the folded RNA strands. Thus, in the case of an rG4 structure formation, only in the presence of K^+^, the increase of cleavage will occur primarily for the nucleotides becoming single-stranded that are located in the loops and the regions immediately upstream and downstream of the rG4. Meanwhile, the G-tracts remain protected.

**Fig 1:**
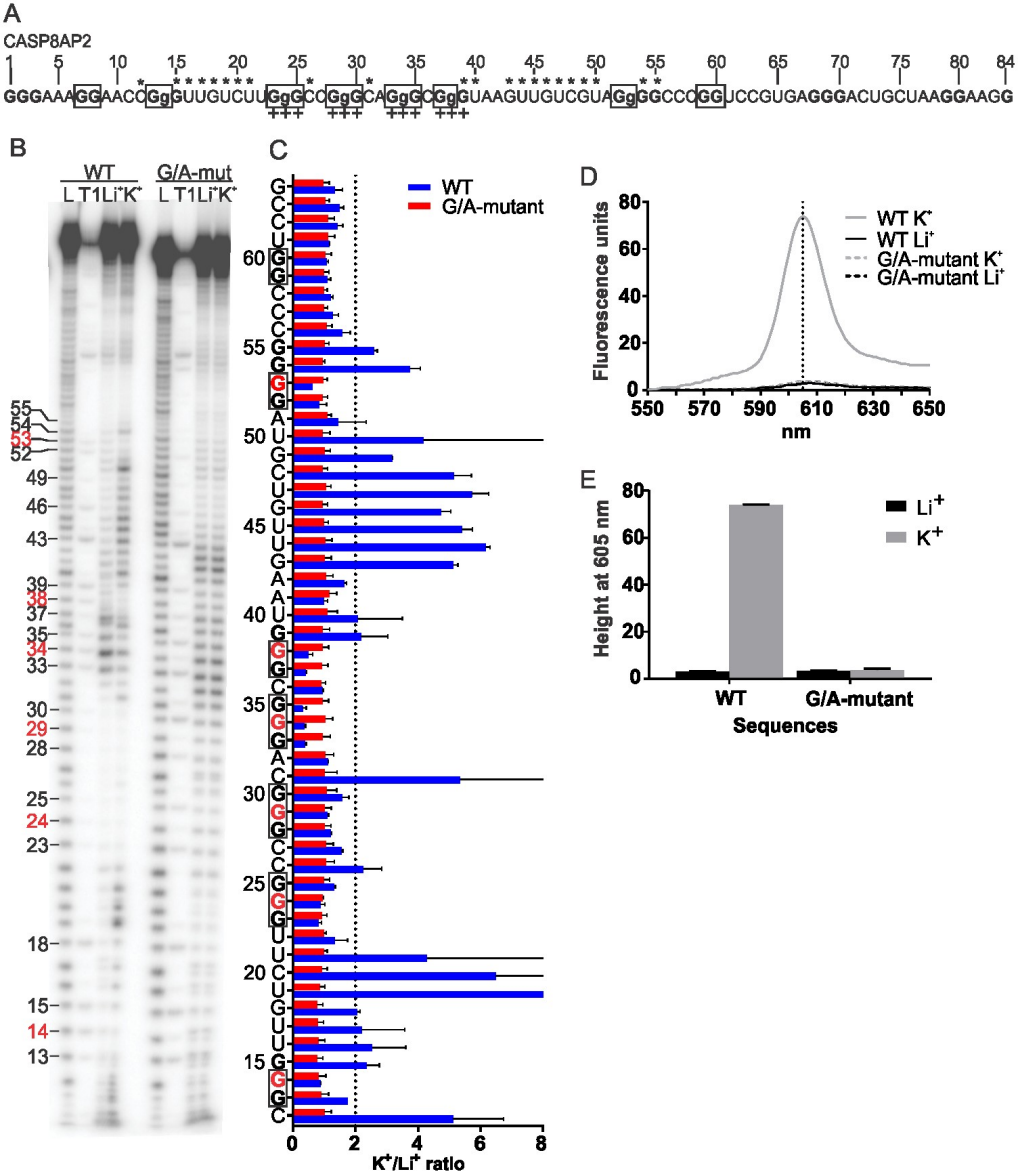
In vitro probing results for the candidate PG4 CASP8AP2. A) Sequence, in the 5’ to 3’ orientation, of the PG4 with the G-tracts in bold and the G mutated to A in lower-case. Asterisks over the sequence indicate nucleotides for which the K^+^/Li^+^ cleavage ratio is higher than the threshold of 2. The “+” symbols indicate the Gs involved in G-tract formation as predicted by the RNAfold algorithm. The boxed G-tracts are those involved in G-quadruplex formation based on the *in-line* probing results. B) Representative phosphorimaging of a CASP8AP2 *in-line* probing denaturing PAGE gel. The alkaline hydrolysis ladder (L) and RNAse T1 ladder (T1) indicate the positions of every nucleotide and every guanine, respectively. Guanine numbering positions are indicated on the left, and the positions in red are those mutated to A in the G/A-mutant. C) K^+^/Li^+^ quantification of the *in-line* probing band intensities for each nucleotide for both the WT sequence (blue) and the G/A-mutant sequence (red). Each bar represents the mean of 2 independent experiments, and the error bars represent the standard deviations. The K^+^/Li^+^ cleavage ratio threshold of 2 is indicated by the dotted line. D) Fluorescence emission curves of the WT and the G/A-mutant RNA sequences of the CASP8AP2 candidate in the presence of NMM after excitation at 399 nm. The full line represents the WT, the dotted line represents the G/A-mutant, the gray line indicate the presence of K^+^ and the black line the presence of Li^+^. Each curve is the mean of 3 independent experiments. The vertical dotted line indicates the 605 nm peak expected when NMM is bound to quadruplex RNA. E) Fluorescence emission peaks observed at 605 nm under the different conditions: Black, Li^+^; Gray, K^+^. Each bar represents the mean of 3 independent experiments, and the error bars are the standard deviations.

A representative example of *in-line* probing is shown in Fig 1 for the Apoptosis related 5’UTR PG4 of the CASP8AP2 mRNA. Following radioactive labelling of the 5’extremity of the *in vitro* transcribed RNA sequence bearing the PG4 motifs and their surrounding nucleotide contexts (Fig 1A), the RNA was heat denatured and allowed to fold by slow cooling. The RNA was then incubated at room temperature for 40 h in the presence of either Li^+^ or K^+^ in order to allow self-cleavage to occur. The RNA was then separated on a denaturing PAGE gel along with the respective alkaline hydrolysis and RNAse T1 sequence ladders so as to be able to identify each nucleotide (Fig 1B). After exposition to a phosphor imaging screen, the density level of each band was measured and compared using the K^+^/Li^+^ cleavage ratio. An arbitrary 2-fold threshold was used to label a nucleotide as being flexible. The K^+^/Li^+^ ratios of the WT and G/A-mutant sequences of CASP8AP2 are presented in Fig 1C. Nucleotides flexible in the presence of K^+^, and thus specific to the rG4 favorable condition, as well as protected G-tracts, are shown on Fig 1A. For the CASP8AP2 candidate, the cleavage pattern of the nucleotides in between G-tracts is representative of an rG4 folding. The presence of more than 4 protected G-tracts indicates the possibility of multiple co-existing rG4 formations using different combinations of loops and G-tracts. The “+” symbols below the nucleotides in positions 23-24-25, 28-29-30, 33-34-35 and 37-38-39 in Fig 1A indicate the G-tracts of the rG4 secondary structure predicted by the RNAfold algorithm. Because the guanine located at position 39 shows a high K^+^/Li^+^ ratio, it was considered as being flexible; hence, it is unlikely that this series of Gs is part of the rG4. Thus, RNAfold positively predicted rG4 formation in the CASP8AP2 sequence, but at the incorrect G-tracts positions.

### rG4 formation was confirmed by an NMM fluorescence assay

In order to further validate the claim of rG4 formation in the CASP8AP2 candidate, a second *in vitro* supporting technique was used. N-Methyl Mesoporphyrin IX (NMM) is a ligand that had previously been shown to specifically bind to DNA G4 with a parallel topology [35]. The ligand by itself emits a very low fluorescence, but upon binding to a parallel G4, its fluorescence can be increased from 2- to 10-fold. As RNA G4 mostly adopt parallel topology because of the *anti*-conformation of the ribose moiety [51], the fluorescent enhancement of NMM in the presence of K^+^ and WT sequences, as compared to that in presence of Li^+^ and G/A-mutant sequences, was used as confirmation of the rG4 folding of the candidates. Fig 1D presents the fluorescent emission curves of NMM after excitation at 399 nm following a 30 min incubation with either the WT or the G/A-mutant sequences of CASP8AP2 under the various Li^+^ and K^+^ conditions. The characteristic peak at 605 nm for parallel G4 binding was observed. The measured fluorescence emission of the 605 nm peak is presented in Fig 1E, with enhancement only being observed in the rG4-prone WT and K^+^ conditions. This result confirms the rG4 folding of the CASP8AP2 candidate observed by *in-line* probing.

### Approximately half of the 5’UTR PG4 sequences do fold *in vitro* into an rG4

**Fig 2:**
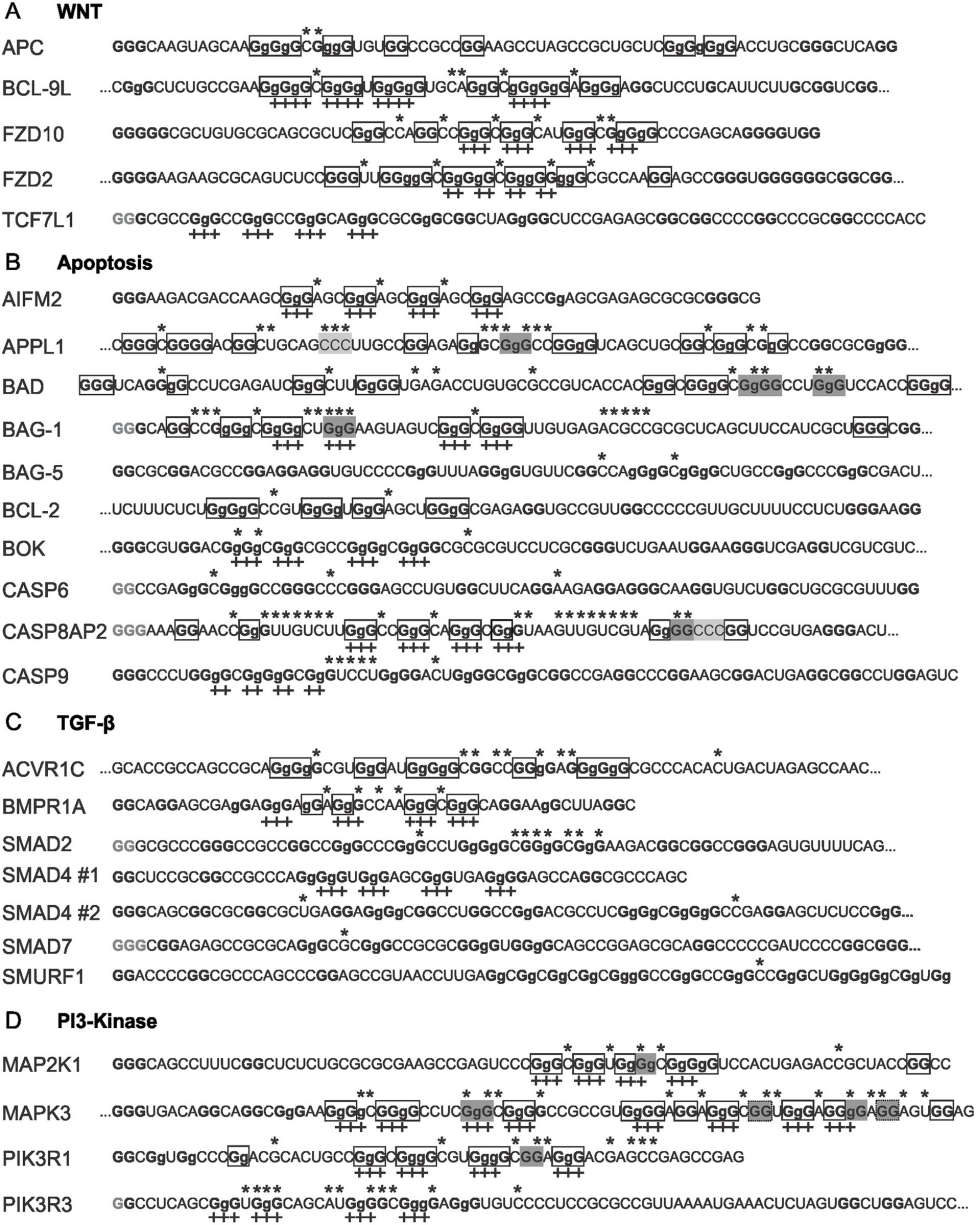
*In-line* probing results for all candidates classified by pathway. A) WNT; B) Apoptosis; C) TGF-β; and, D) PI3-Kinase. The sequences are in the 5’ to 3’ orientation, the series of 2 or more consecutive Gs are in bold and the G mutated to A in the mutants are in lower-case. The presence of three dots at either the beginning or the end of a sequence indicates that the RNA sequence actually tested was longer, but could not be presented fully here due to space limitations. The gray guanines at the 5’ extremity were artificially added in order to optimize the *in vitro* transcription reactions. The full sequences tested are listed in S1 Table. The asterisks over the nucleotides indicate a K^+^/Li^+^ cleavage ratio higher than the threshold of 2. The “+” symbols underneath the sequence indicate the Gs involved in G-tract formation as predicted by the RNAfold algorithm. The boxed G-tracts in white are those involved in G-quadruplex formation based on both the *in-line* probing and the NMM fluorescence assay results. The hatched boxes are the G-tracts alternatively involved in G-quadruplex formation depending on the different G to A mutations (see MAPK3 in S1B Fig). Series of 2 or more consecutives Gs present in the loops are highlighted in dark gray, and series of 3 consecutive Cs in the loops are highlighted in pale gray.

*In-line* probing of 19 candidates was performed, and the results were supplemented with the already available *in-line* probing data of the candidates APC from one study [24] and FZD2, TCF7L1, ACVR1C, SMAD2, SMAD7 and MAPK3 from another [29]. Representative *in-line* gels with quantifications from two independent experiments for all of the candidates are available in S1 Fig. The NMM fluorescence assay was performed for all 26 PG4 sequences. The results are presented in S2 Fig. The results of both *in vitro* techniques are summarized in Table 2. The rG4 formation was confirmed for 15 candidates, 9 of them for the first time (BCL-9L, FZD10, AIFM2, APPL1, BAD, CASP8AP2, BMPR1A, MAP2K1 and PIK3R1). NMM fluorescence assays confirmed the previous conclusions of rG4 folding of APC, FZD2, BAG-1, BCL-2, ACVR1C and MAPK3, and the dsRNA folding of TCF7L1, SMAD2 and SMAD7. The dsRNA folding of the SMURF1 candidate observed here both by *in-line* probing and NMM fluorescence assay is different from that of a prior CD study [52] which concluded that there was rG4 formation. In this prior study, only the region corresponding to positions 49 to 66 of the 100 nts sequence probed here was used. The presence of a biologically relevant competitive nucleotide context in the present study might explain the discrepancy between the 2 studies. The folding of a dsRNA structure was also observed for BAG-5, BOK, CASP6, CASP9, the two sequences from SMAD4 and PIK3R3. Fig 2 presents the sequences probed for all of the 5’UTR PG4s of the mRNAs, classified by their associated pathway. The flexible nucleotides and protected G-tracts are identified. The PG4 sequences that were assigned positive for rG4 formation are presented with boxed G-tracts.

Overall, the two experimental techniques used to evaluate rG4 or dsRNA folding were in good agreement. The *In-line* probing pattern of the cleavage representative of rG4 folding under K^+^ conditions was generally associated with a high K^+^ versus Li^+^ difference in the 605 nm fluorescence peak enhancement (Table 2). The average difference value of the NMM 605 nm fluorescence peak was 16.5 ± 16.2 (mean ± standard deviation) for dsRNA and 58.4 *±* 18.8 for rG4 folding. Because of the extent of variation in the NMM fluorescence, when the difference between the K^+^ and Li^+^ fluorescence emission peaks was intermediate (i.e. ~ 30) preponderance was given to the *in-line* probing results in the final assignment of rG4 (+) or dsRNA (-) structure formation. For examples, the BAD candidate presented an rG4 folding pattern using its *in-line* probing results, but a modest fluorescent enhancement and was assigned positive for rG4 folding. On the other hand, the CASP9 candidate did not presented a convincing rG4 pattern using *in-line* probing despite high fluorescence in presence of NMM and was assigned negative. However, it is not excluded that sequences with apparent contradictory results could adopt either folding types in different conditions than the ones assessed here. Variation in the fluorescence enhancement from one rG4 candidate to another could be explained by different rG4 features, such as both the G-tracts’ numbers and sizes and the loops’ sizes and compositions. The variation could also be explained by the proportion of RNA strands that folded as an rG4 versus as a dsRNA (e.g. the equilibrium between the competing structures) differing for each candidate. The results of the *in vitro* confirmation of rG4 folding demonstrate that many sequences possessing the consensus PG4 sequence motif prefer to adopt a dsRNA structure. This result is similar to that of a previous study of PG4 sequences [6]. The rG4 prediction based on motif search only is unreliable. Thus, it is essential to confirm the folding with multiple reliable experimental techniques, and to use different prediction tools that take into consideration the competing nucleotide context.

### G4NN is the most accurate predictor of in vitro rG4 formation for this set of PG4

With both sets of experimental results in hand, confirming or not rG4 formation, it is possible to compare the accuracy of the rG4 prediction tools. S2 Table presents the numbers of all true positive and negative predictions for each of the three scores, as well as for the RNAfold algorithm. These values were used to calculate the sensitivity and specificity of each predictor. G4NN had the highest number of true positive and true negative predictions, giving it the best combination of high sensitivity and high specificity of all predictors. The cG/cC score and RNAfold have similar levels of sensitivity and specificity, but had higher numbers of false positives than did G4NN. As is observable by the comparison of the G-tracts with the “+” sign predicted by RNAfold with those actually observed by *in-line* probing (boxed G-tracts in Fig 2), RNAfold often correctly predicted rG4 folding, but with wrong G-tracts. G4H was originally developed for DNA sequences, which could explain its lower sensitivity for RNA PG4 sequences. However, it showed high specificity, identifying only real rG4, just not all of them. Of the 26 candidates, only 7 were correctly predicted by all of the predictors (rG4: FZD10, FZD2, BMPR1A and MAPK3, dsRNA: BAG-5, SMAD2 and SMAD7). These well-predicted rG4 shared the characteristics of having short G-tracts that are separated by short loops of the same sizes (Fig 2). The well predicted dsRNA candidates presented longer loops and possessed a higher number of consecutive cytosines in order to compete with rG4 formation, and this was well detected by all of the predictors. Conversely, four candidates were wrongly predicted by all of the predictors (CASP9, BAD, BCL-2 and APPL1). These sequences presented larger loop sizes and many G-tracts of various lengths (Fig 2). These features still represent challenges that will need to be addressed for accurate prediction of rG4 folding motifs. The assessment of folding with a technique such as *in-line* probing permits the identification not only of the global rG4 or dsRNA structures, but also of which exact nucleotides are base-paired or not. Consequently, the experimental validation of folding remains essential to observe the limits of the actual prediction tools.

### Most rG4 possess features different than the canonical motif

The *in-line* probing cleavage patterns permit the identification of an rG4 region in a given sequence with all of the possible combinations of four protected G-tracts and three loops with flexible nucleotides (Fig 2). However, this pattern represents the sum of all of the rG4 conformations in solution, and cannot identify which one is the most stable or dominant. S1B Fig presents the *in-line* probing results of different G/A-mutant constructions for the MAPK3 sequence that can support the claim of two consecutive rG4 folding units like the beads-on-string model [26]. Considering all candidates, the majority present more than four G-tracts. This indicates that multiple combinations of G-tracts can be adopted in order to fold into a single rG4. To avoid selecting a “preferred conformation”, all possible G-tracts located immediately 5’ or 3’ of nucleotides with a K^+^/Li^+^ cleavage ratio higher than the threshold of 2 were boxed in the Fig 2. Each supplementary G-tract, adds numerous possibilities of different G-tract combinations. For example, the BAD candidate possesses seven G-tracts, giving rise to 35 possible combinations for rG4 formation.

The sizes of the G-tracts are an important feature of rG4, as the number of Gs comprising the G-tracts represents the number of stacked quartets of the resulting intramolecular quadruplex. The WNT set of positive rG4 candidates presents a lower number of G‑tracts composed of 2Gs, and a higher number of G-tracts with 5Gs or more, in comparison with both the Apoptosis, the TGF-β and the PI3-K sets. As expected, the majority of the G-tracts are 3Gs in size as was defined in the initial sequence motif search. Interestingly, less than half of the positive rG4 candidates possessed four G-tracts of the same size. This means that the G-quartets of the structure are formed from G-tracts of various lengths, and that either not all of the Gs from the same tract are used simultaneously, or bulges might be present. Again, this result demonstrates why sequence motif searches and rG4 predictions based primarily on finding sequences with four identical lengths of G-tracts is not accurate.

The second most important feature that is required in order to define an rG4 is the loops linking the G-tracts. By broadly defining the rG4 loops as the nucleotides linking the protected G-tracts from the *in-line* probing cleavage pattern in the K^+^ condition, one can observe that the rG4 candidates present loops that can vary greatly from the size of 1 to 7 nt defined in the canonical rG4 motif. The APPL1, BAD, BAG-1, CASP8AP2 and MAPK3 candidates *in-line* cleavage patterns allow for possible loops larger than 7 nts (Fig 2). Another distinctive feature of the loops is their nucleotide composition. No differences were observed in the ratios of A, U, C and G nucleotides in the loops. However, the presence of three consecutives Cs or Gs, in the loops, which could be respectively detrimental or beneficial for rG4 formation is highlighted in Fig 2.

These specific features, such as the sizes of the G-tracts and the sizes of the loops, might affect the stabilities of the different rG4s. For example, a stack of four quartets is more stable than a stack of three, and shorter loops are more stable than longer ones. Moreover, a combination of distinct features (G-tract numbers and sizes; loop numbers, sizes and composition) could also serve as motifs for recognition by *trans-*factors or helicases. The WRN DNA G4 helicase has been shown to bind G4s that are located in promoters. The helicase targets G4s that possess specific features that are different from those recognized by another DNA G4 helicase, specifically BLM [53]. Consequently, a similar recognition of specific features for rG4-helicase is a possibility. The development of chemical ligands with which to specifically target the rG4 structures is now a thriving field. The identification of specific features allows one to steer the design of a ligand such that it can target specific subsets of rG4 more precisely.

### rG4 folding affects the expression level of a luciferase reporter gene in colorectal cancer cells

The *in vitro* experiments confirmed that rG4s are folded in the 5’UTR of many mRNAs involved in dysregulated colorectal pathway, suggesting a possible regulatory role for the structure. However, the *in cellulo* impact of the structure on the regulation of gene expression cannot be directly inferred from evidence of *in vitro* formation. In order to evaluate the potential role of the 5’UTR rG4s on mRNA regulation of expression, three selected rG4 candidates associated to different pathways (BAG-1 and CASP8AP2 for Apoptosis and MAPK3 for PI3‑K) were selected for a gene reporter assay [54]. These candidates were selected in order to have at least one representative candidate for each pathway enriched with rG4 forming sequences. The TGF-β pathway was not further considered as “enriched” for rG4 structure because it contains only 2 positive rG4s out of 7 sequences. The candidates were the ones with the highest scores for each of the four *in silico* prediction tools and clear *in-line* probing and NMM fluorescence confirmations of rG4 formation. Importantly, these candidates have not been previously tested *in cellulo*. To ease the cloning procedures, the selected candidate also possessed short 5’UTR sequence. The three selected candidates were compared for their *in cellulo* effect on luciferase expression with candidates from the WNT (APC and FZD2) and the Apoptosis (BCL-2) pathways already evaluated in previous work [6,24,47].

In order to perform the *in cellulo* assay, the entire 5’UTR containing the rG4 of the candidate was inserted upstream of a *Renilla Luciferase* (*Rluc*) reporter gene, and the resulting construct was then transfected into HEK293 cells. In parallel, the full length 5’UTR, bearing the same G/A-mutations confirmed to be negative for rG4 formation in the *in vitro* assays was also transfected in order to compare the effect of rG4 abolition on the expression level. A second plasmid coding for the *Firefly Luciferase* (*Fluc*) was co-transfected for normalization purposes. For all four candidates the *Rluc* normalized expression level was higher, with an approximately 2-fold increase for the G/A-mutants, in which rG4 formation was abolished, as compared to the WT (S3 Fig). The formation of an rG4 in these four candidates represses expression, as was observed previously for many other rG4s located in the 5’UTR [54,55].

The *in-line* probing results suggests that the MAPK3 candidate formed two adjacent rG4s. In order to confirm this observation, different G/A-mutants where designed so as to abolish either the first possible rG4, the second, or both of them. As seen in S3D Fig, the two G/A‑mutants impairing a single rG4 have similar *Rluc* normalized expression levels that are almost 3-fold higher than that of the WT sequence, but the double rG4 mutant exhibits a 6-fold increase in the expression level of the *Rluc* reporter gene over that of the WT. This indicates that there is an additive, repressive effect of the two rG4. Even with the double rG4 mutant, in which 9 Gs were mutated to As, not all possible rG4 formations were abolished. The *in-line* probing (S1B Fig) and NMM fluorescence assay (S2 Fig) results of MAPK3 demonstrated that the double G/A‑mutant can still adopt an rG4, albeit possibly a less stable one possessing only 2 stacked quartets and loops sizes of 3 and 5 nts. Thus, the 6-fold increase in the expression level of the double rG4 mutant over the WT observed *in cellulo* might actually be higher if all potential rG4 were eliminated.

Gene-reporter assays using HEK293 cells permit comparisons with previous studies in which rG4 located in 5’UTRs were evaluated for their repressive effects on expression levels *in cellulo*. The fold changes of the normalized luciferase expression of the mutant over WT (2.40-fold for BAG-1 and 2.07-fold for CASP8AP2) are in the same range as the ones observed for the APC, BCL-2 and FZD2 rG4s in earlier studies (1.74-fold for APC [24], 2.30 -fold for BCL-2 [47] and 2.50-fold for FZD2 [6]). However, because the mRNA 5’UTR rG4 candidates selected here were associated with colorectal cancer dysregulated pathways, the gene-reporter assays were also performed using three representative colorectal cell lines: HCT116, HT29 and DLD-1 for the APC, BAG-1 and CASP8AP2 candidates (S4 Fig). The MAPK3 candidate was not further tested in colorectal cell lines, as no complete rG4 negative control was possible without highly mutating the short 5’UTR sequence with supplementary G-to-A mutations.

In general, the results replicate what was observed with the HEK293 cells. In the colorectal cell lines the normalized expression of the *Rluc* reporter gene was higher when the rG4 was mutated for all three of the candidates tested. Despite slight differences in the expression levels, the mutant over WT fold-changes were very similar for a specific candidate between the three cells lines. The APC G/A-mutant fold-change was ~2 (2.09-fold in HCT116, 2.11-fold in HT29 and 2.65-fold in DLD-1) and the BAG-1 fold-change was ~3 (3.56-fold in HCT116, 3.11-fold in HT29 and 3.92-fold in DLD-1). The difference was not statistically significant for the CASP8AP2 candidate, which showed an almost 2-fold increase in the expression level for the mutant in all three cell lines (1.87-fold in HCT116, 1.95-fold in HT29 and 2.01-fold in DLD-1). In brief, rG4 folding in the 5’UTR of these mRNAs associated with colorectal cancer dysregulated pathways represses the expression level of the luciferase reporter gene in the relevant colorectal cancer cells models.

The *in cellulo* assays performed cannot decipher at which level, transcriptional, post-transcriptional or both, the regulation occurred. However, based on actual knowledge of rG4 regulation in 5’UTRs [14], translational repression seems to be the probable mechanism. The range of repression levels between the different rG4 candidates, and between the different cell lines, observed here and in other studies is quite narrow, generally being a 2-to-3 fold-change between the WT rG4 and the mutated sequence. Nevertheless, the different levels of repression observed between the candidates might be explained by how the different features of the rG4s themselves (i.e. the G-tracts and loops) affect their stabilities, and also by the differences in their full 5’UTR contexts (i.e. the position where the rG4 is located in the 5’UTR and exactly what are the adjacent sequences). Bhattacharyya *et al*. [56] analyzed the role of the context by interchanging two rG4s that exhibited opposite effects on expression (one was an enhancer, the other a repressor). After the switch, the rG4 in the new position mimics the effect on expression level of the original rG4. It showed that the context is also responsible for the rG4 mechanism of regulation. For a particular rG4 candidate in its natural 5’UTR context, the differences in the levels of repression between the alternate cell lines might be caused by variations in the *trans-*factors that are expressed in those cell lines and that take part in the rG4-mediated regulation of mRNA expression. In order to continue with the hypothesis that rG4s located in the 5’UTR are part of a global regulation mechanism, it would be interesting to compare the characteristics of the 5’UTR in which they are found for other similarities (position of the rG4, upstream ORFs, IRES, translation regulatory sequences, protein-binding motifs, etc.) that could also be specific to each pathway.

## Conclusion

The enrichment of rG4 prone sequences in the 5’UTRs of mRNAs, and their known repressive effects on translation, point towards a possible role for these structures in the global regulation of mRNAs involved in common biological pathways. This study showed that mRNAs bearing a consensus PG4 sequence in their 5’UTRs are enriched in some of the KEGG annotated pathways, for example in the general colorectal cancer pathway. *In vitro* evaluation of the folding of 26 selected PG4 candidates associated with well-defined colorectal cancer dysregulated pathways confirmed rG4 folding for 15 of them and *in cellulo* reporter assays using colorectal cell lines demonstrated their effect on mRNA expression level for 3 of them.

This study adds new, experimentally confirmed sequences to the list of rG4s located in the 5’UTR that could affect gene expression. It demonstrates that rG4 prediction based solely on a sequence motifs search is insufficient. The available *in silico* prediction tools, such as G4NN which was the best one for the set of candidates examined here, can improve the selection of rG4 prone sequences, but cannot yet correctly predict which sequences will fold, nor which exact nucleotides of the sequence are involved in the structure.

*In-line* probing of rG4 sequences permits identification of the nucleotides involved in quadruplex formation, and thus comparison of their features (e.g. G-tract numbers, sizes, loops sizes and compositions). However, further studies are needed in order to uncover specific rG4 features shared by mRNAs involved in a common biological pathway, and to better understand the role of both helicases and rG4 binding proteins in the recognition mechanism of subsets of distinct rG4s.

## Supporting information

S1 Fig *In-line* probing gels and K^+^/Li^+^ ratio quantification of the candidates. Representative phosphorimaging of *in-line* probing denaturing PAGE and K^+^/Li^+^ ratio quantification of the band densities for each nucleotide for both the WT sequence (blue) and the G/A-mutant sequence (red). The results are presented in the alphabetical order of the candidates’ names. A) ACVR1C, AIFM2, APC, APPL1, BAD, BAG-1, BAG-5, BCL-2, BCL-9L, BMPR1A, BOK, CASP6, CASP8AP2, CASP9, FZD2, FZD10, MAP2K1, PIK3R1, PIK3R3, SMAD2, SMAD4 #1, SMAD4 #2, SMAD7, SMURF1 and TCF7L1. The alkaline hydrolysis ladder (L) and RNAse T1 ladder (T1) indicate the positions of every nucleotide and every guanine, respectively. Guanine numbering positions are indicated on the left, and the positions in red are those mutated to A in the G/A-mutant. On the quantification graph, each bar represent the mean of at least 2 independent experiments, and the error bars represent the standard deviations. The K^+^/Li^+^ ratio threshold of 2 is indicated by the dotted line. B) Details of the *in‑line* probing results for the MAPK3 candidate and its different mutated sequences. *Upper panel*: sequences of the MAPK3 WT candidate and its various G/A-mutants that were tested. The last sequence summarizing the results is also presented as in Fig 2. The asterisks over the sequence indicate nucleotides for which the K^+^/Li^+^ cleavage ratio was higher than the threshold of 2. The boxed G-tracts are those involved in G-quadruplex formation. The hatched boxes are the G‑tracts alternatively involved in G-quadruplex formation depending on the different G to A mutations. *Lower panel*: *In-line* probing denaturing PAGE and the K^+^ /Li^+^ ratio quantification for the MAPK3 WT candidate and its different G/A mutants. The WT results are in blue, the double G/A‑mut results are in red, the 1^st^ G/A-mut results are in green and the 2^nd^ G/A-mut results are in purple.
(PDF)

S2 Fig NMM assay of all candidates. The fluorescence emission peaks at 605 nm under the different conditions: Black Li^+^, Gray K^+^. Each bar represents the mean of 3 independent experiments and the error bars represent the standard deviations.
(PDF)

S3 Fig *In cellulo* luciferase assay in HEK293 cells. Results for A) BAG-1 and B) CASP8AP2 from the Apoptosis set; and C) MAPK3 from the PI3-K set. The results are shown as the means of the Rluc expression normalized over the Fluc transfection control. The WT results are in black and the G/A‑mutants are in different shades of gray. The error bars represent the standard deviations. Statistical difference was measured using an unpaired Student t-test with a n = 2 for APC, n = 3 for BAG-1 and CASP8AP2 and n = 5 for MAPK3. *P-value < 0.05 **P-value < 0.01 ***P-value < 0.001.
(PDF)

S4 Fig *In cellulo* luciferase assay in colorectal cancer cell lines. The WT and the G/A-mutant full-length 5’UTRs were inserted upstream of the Renilla luciferase (Rluc) reporter gene and used for transfection. The G mutated to A were the same as those in the *in vitro* assays. A) APC, B) BAG-1 and C) CASP8AP2. The results are shown as the means of the Rluc expression normalized over the Fluc transfection control in the three colorectal cell lines HCT116, HT29 and DLD-1. The WT results are in black and the G/A-mutants’ results are in gray. The error bars represent the standard deviations. Statistical difference was measured using an unpaired Student t-test with a n = 3 *P‑value < 0.05 **P-value < 0.01.
(PDF)

S1 Table Sequences, positions in the 5'UTR and lengths of all candidates and their respective full-length 5'UTRs. (XLSX)

S2 Table Comparison of the prediction methods. (PDF)

S3 Table UTRref, RefSeq and gene-ontology identification numbers of all candidates. (XLSX)

S4 Table Oligonucleotide sequences used for PCR-filling prior to in vitro transcription. (XLSX)

S5 Table Oligonucleotide sequences used for PCR filling prior to cloning. (XLSX)
