# Supplementary material for: G-quadruplexes formation in the 5’UTRs of mRNAs associated with colorectal cancer pathways
Source: PLoS One. 2018 Dec 3;13(12):e0208363. doi: 10.1371/journal.pone.0208363 (PMC6277105; doi:10.1371/journal.pone.0208363)
Supplement: S1 Fig — Representative phosphorimaging of in-line probing denaturing PAGE and K+/Li+ ratio quantification of the band densities for each nucleotide for both the WT sequence (blue) and the G/A-mutant sequence (red). The results are presented in the alphabetical order of the candidates’ names. A) ACVR1C, AIFM2, APC, APPL1, BAD, BAG-1, BAG-5, BCL-2, BCL-9L, BMPR1A, BOK, CASP6, CASP8AP2, CASP9, FZD2, FZD10, MAP2K1, PIK3R1, PIK3R3, SMAD2, SMAD4 #1, SMAD4 #2, SMAD7, SMURF1 and TCF7L1. The alkaline hydrolysis ladder (L) and RNAse T1 ladder (T1) indicate the positions of every nucleotide and every guanine, respectively. Guanine numbering positions are indicated on the left, and the positions in red are those mutated to A in the G/A-mutant. On the quantification graph, each bar represent the mean of at least 2 independent experiments, and the error bars represent the standard deviations. The K+/Li+ ratio threshold of 2 is indicated by the dotted line. B) Details of the in‑line probing results for the MAPK3 candidate and its different mutated sequences. Upper panel: sequences of the MAPK3 WT candidate and its various G/A-mutants that were tested. The last sequence summarizing the results is also presented as in Fig 2. The asterisks over the sequence indicate nucleotides for which the K+/Li+ cleavage ratio was higher than the threshold of 2. The boxed G-tracts are those involved in G-quadruplex formation. The hatched boxes are the G‑tracts alternatively involved in G-quadruplex formation depending on the different G to A mutations. Lower panel: In-line probing denaturing PAGE and the K+ /Li+ ratio quantification for the MAPK3 WT candidate and its different G/A mutants. The WT results are in blue, the double G/A‑mut results are in red, the 1st G/A-mut results are in green and the 2nd G/A-mut results are in purple. (PDF) [file pone.0208363.s001.pdf]

Figure S1 (Jodoin & Perreault, 2018)

A

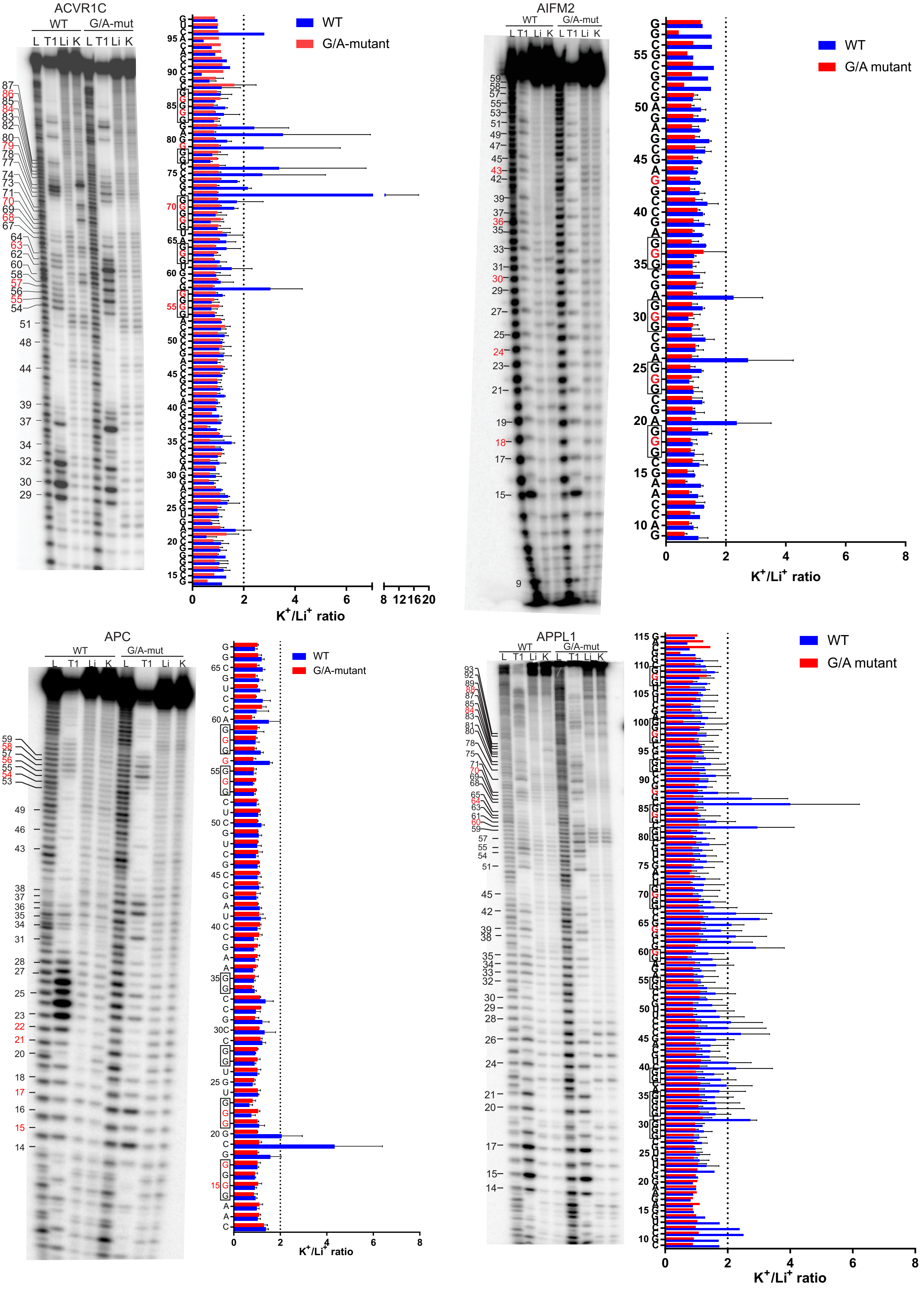

Figure S1*A* (Jodoin & Perreault, 2018)

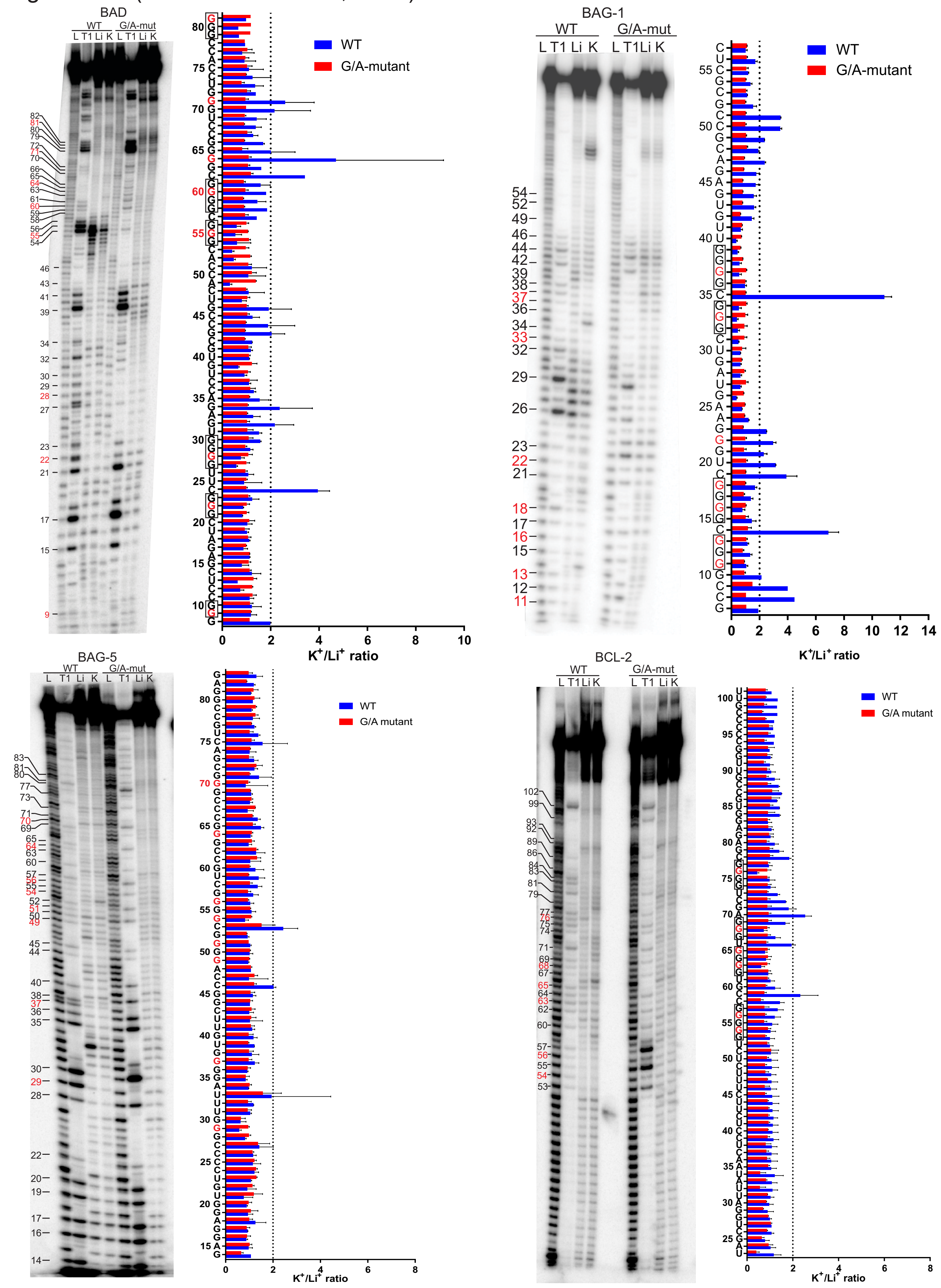

Figure S1*A* (Jodoin & Perreault, 2018)

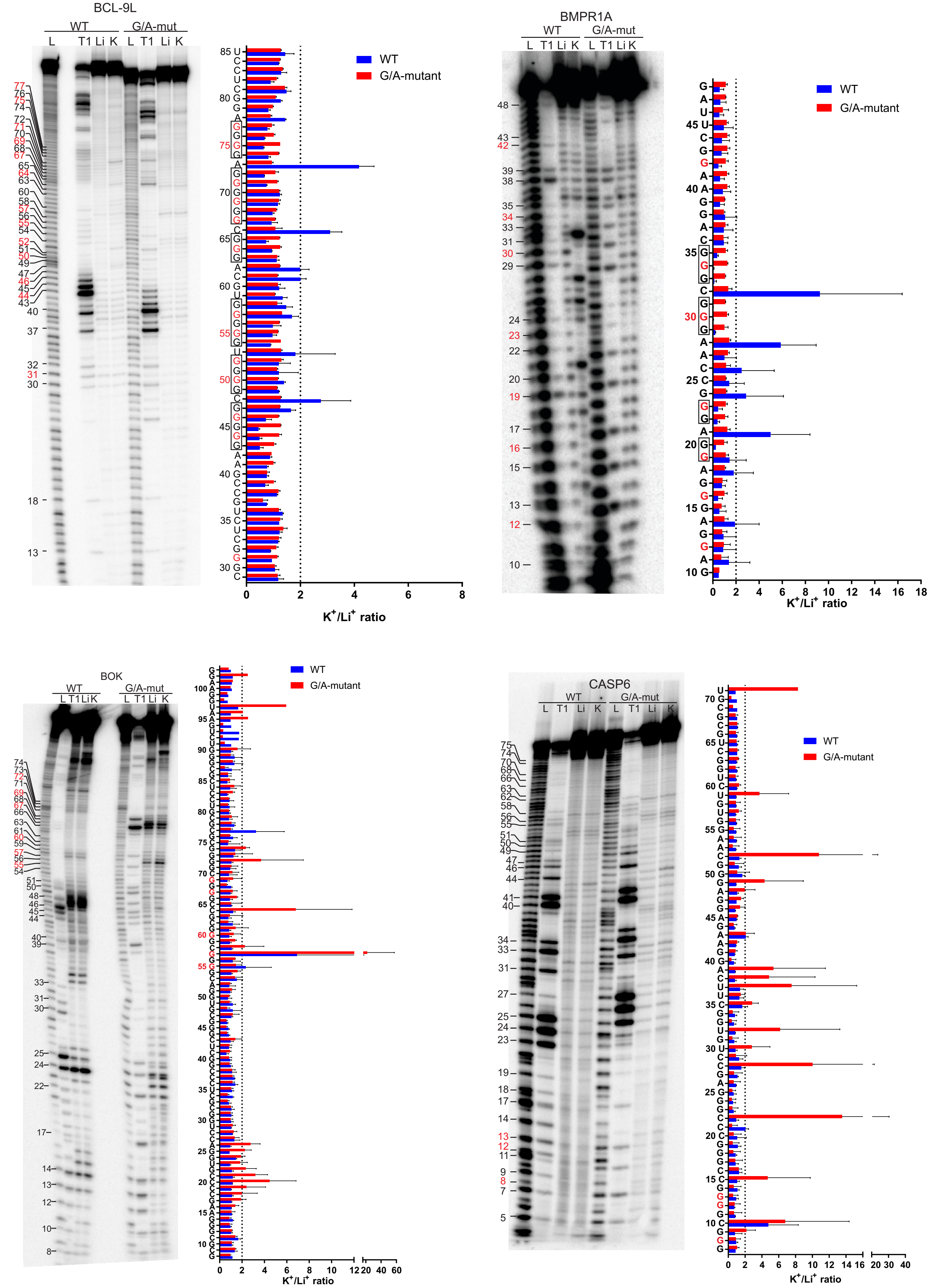

Figure S1*A* (Jodoin & Perreault, 2018)

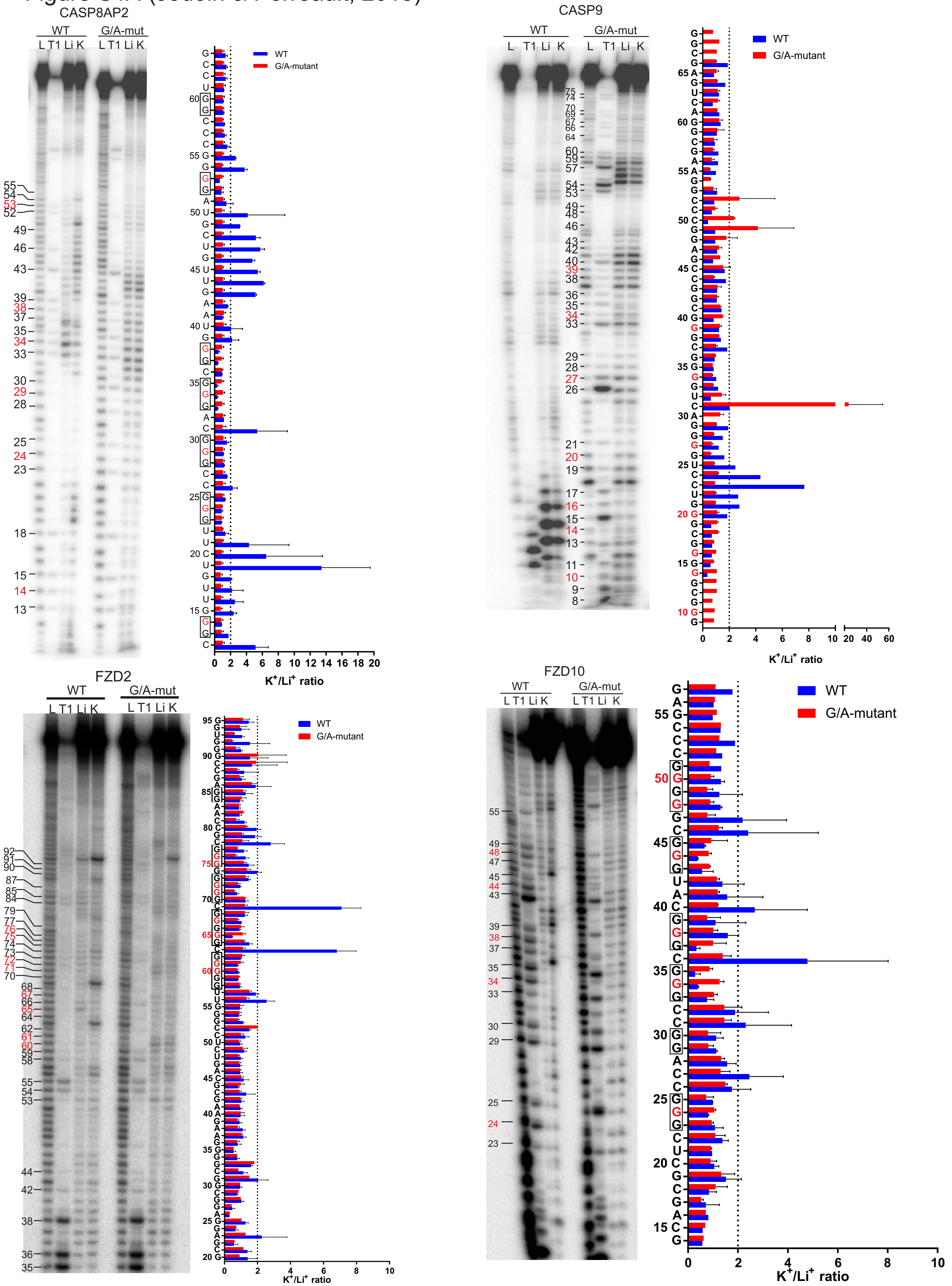

Figure S1*A* (Jodoin & Perreault, 2018)

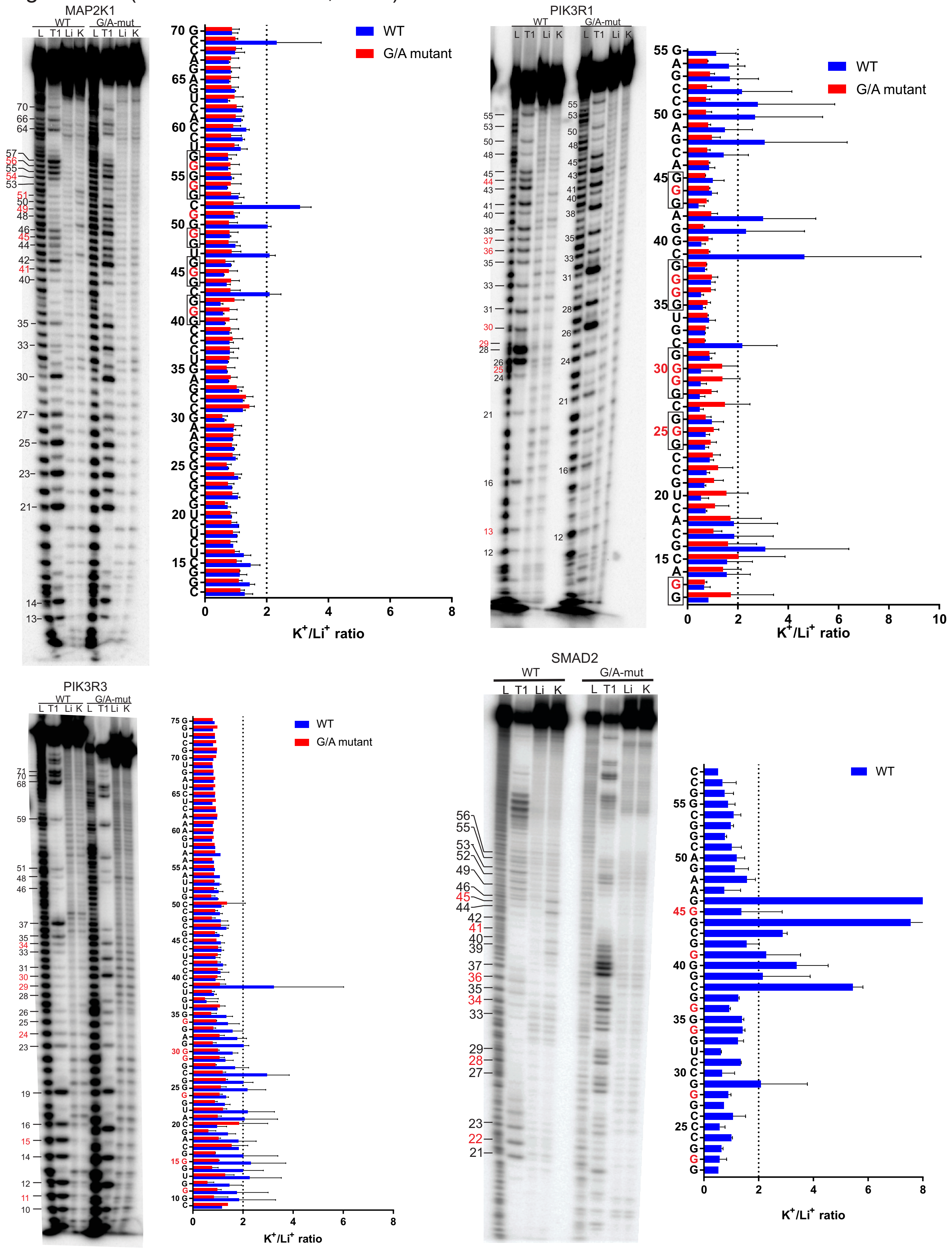

Figure S1*A* (Jodoin & Perreault, 2018)

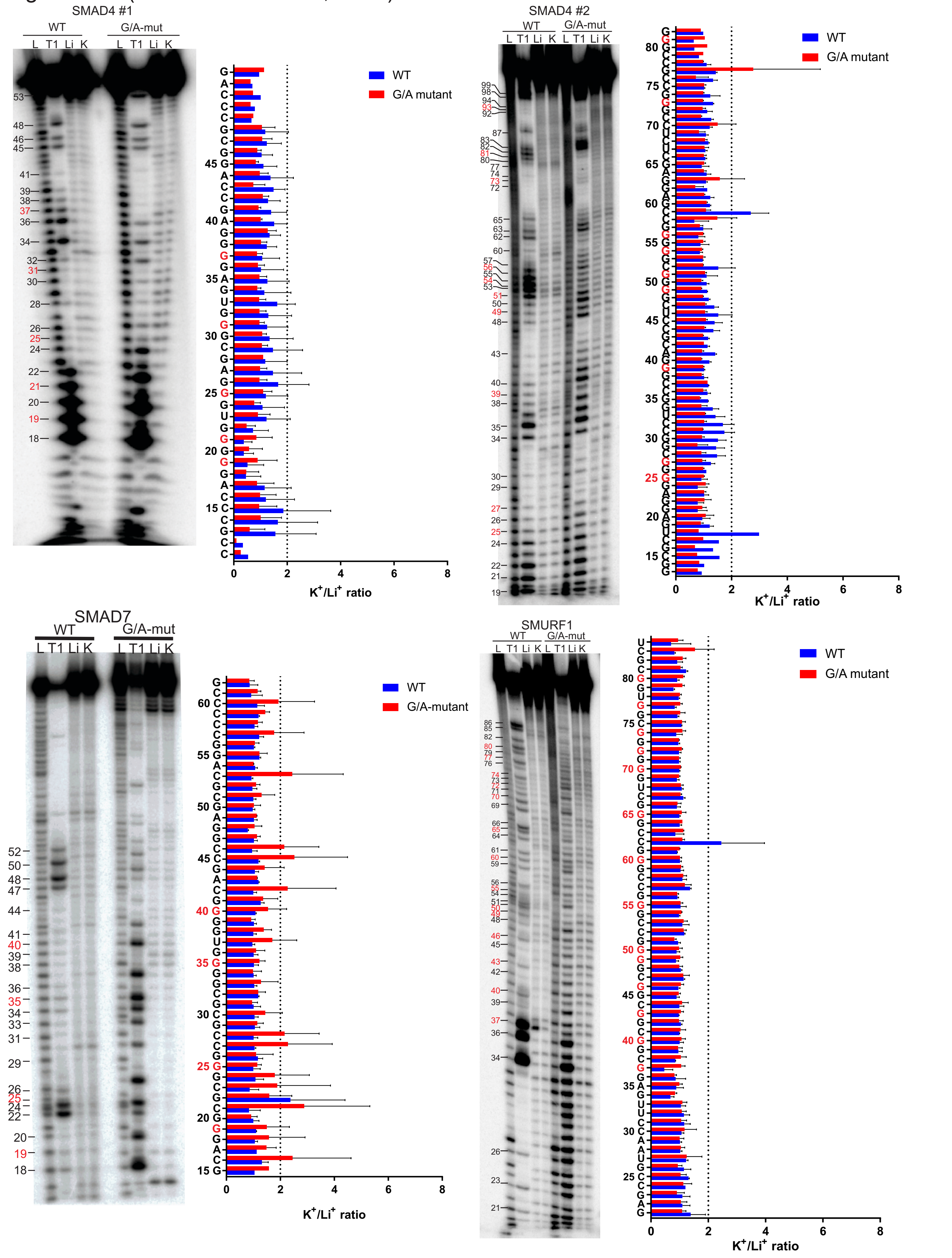

Figure S1*A* (Jodoin & Perreault, 2018)

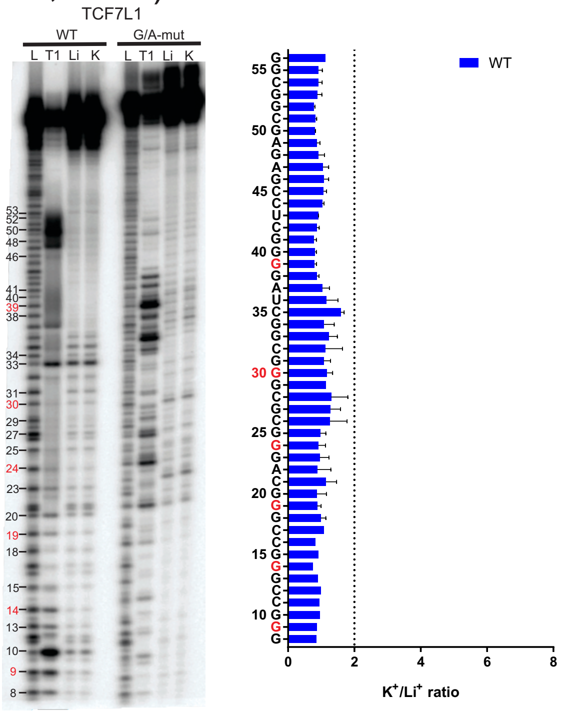

B

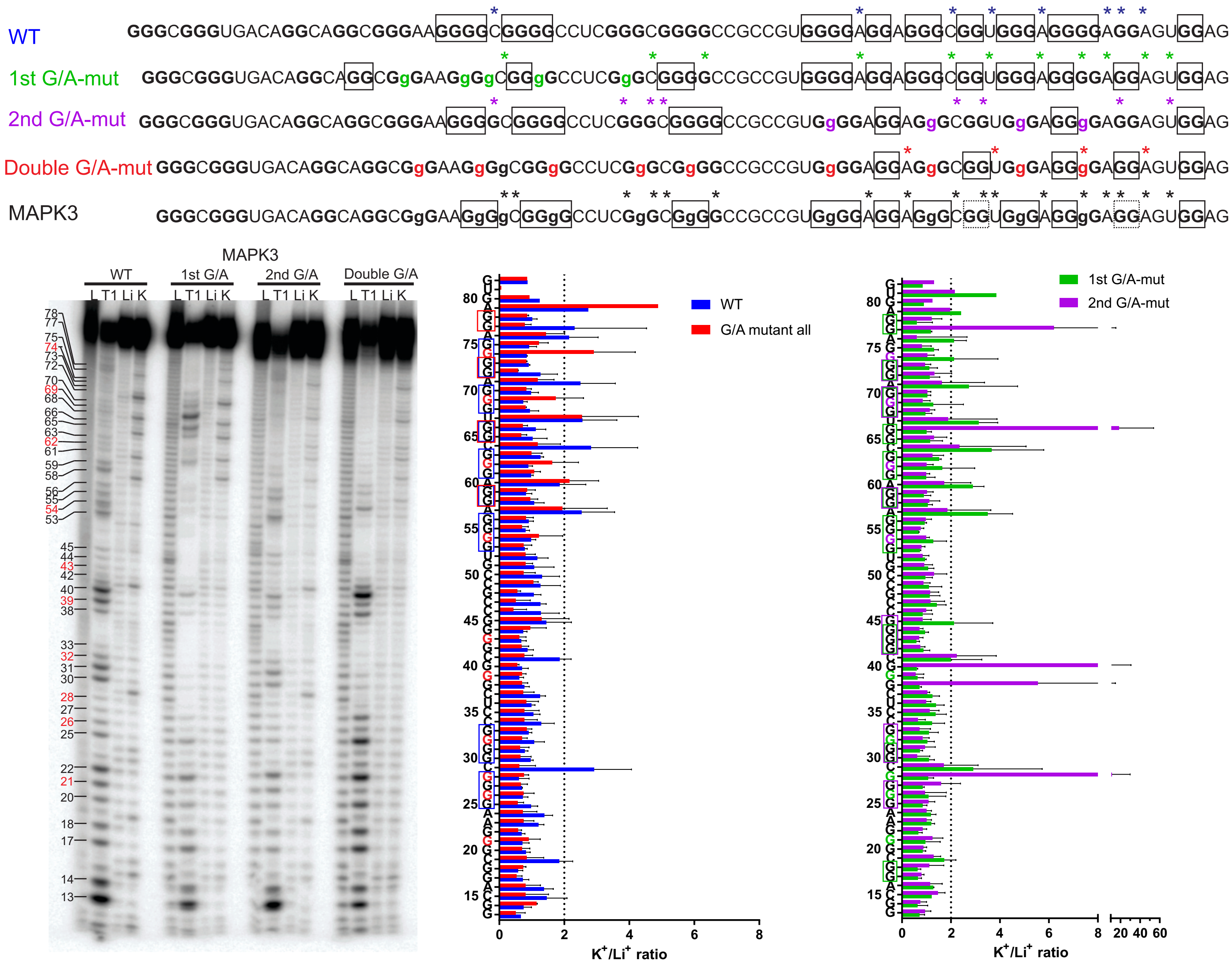

Figure S1 *In-line* probing gels and K^+/Li^+ ratio quantification of the candidates

Representative phosphorimaging of *in-line* probing denaturing PAGE and K^+/Li^+ ratio quantification of the band densities for each nucleotide for both the WT sequence (blue) and the G/A-mutant sequence (red). The results are presented in the alphabetical order of the candidates' names. A) ACVR1C, AIFM2, APC, APPL1, BAD, BAG-1, BAG-5, BCL-2, BCL-9L, BMPR1A, BOK, CASP6, CASP8AP2, CASP9, FZD2, FZD10, MAP2K1, PIK3R1, PIK3R3, SMAD2, SMAD4 #1, SMAD4 #2, SMAD7, SMURF1 and TCF7L1. The alkaline hydrolysis ladder (L) and RNAse T1 ladder (T1) indicate the positions of every nucleotide and every guanine, respectively. Guanine numbering positions are indicated on the left, and the positions in red are those mutated to A in the G/A-mutant. On the quantification graph, each bar represent the mean of at least 2 independent experiments, and the error bars represent the standard deviations. The K^+/Li^+ ratio threshold of 2 is indicated by the dotted line. B) Details of the *in-line* probing results for the MAPK3 candidate and its different mutated sequences. *Upper panel:* sequences of the MAPK3 WT candidate and its various G/A-mutants that were tested. The last sequence summarizing the results is also presented as in **Fig. 2**. The asterisks over the sequence indicate nucleotides for which the K^+/Li^+ cleavage ratio was higher than the threshold of 2. The boxed G-tracts are those involved in G-quadruplex formation. The hatched boxes are the G-tracts alternatively involved in G-quadruplex formation depending on the different G to A mutations. *Lower panel: In-line* probing denaturing PAGE and the K^+ $/Li^+$ ratio quantification for the MAPK3 WT candidate and its different G/A mutants. The WT results are in blue, the double G/A-mut results are in red, the 1st G/A-mut results are in green and the 2nd G/A-mut results are in purple.
