# Supplementary material for: G-quadruplexes formation in the 5’UTRs of mRNAs associated with colorectal cancer pathways
Source: PLoS One. 2018 Dec 3;13(12):e0208363. doi: 10.1371/journal.pone.0208363 (PMC6277105; doi:10.1371/journal.pone.0208363)
Supplement: S2 Fig — The fluorescence emission peaks at 605 nm under the different conditions: Black Li+, Gray K+. Each bar represents the mean of 3 independent experiments and the error bars represent the standard deviations. (PDF) [file pone.0208363.s002.pdf]

Figure S2 (Jodoin & Perreault 2018)

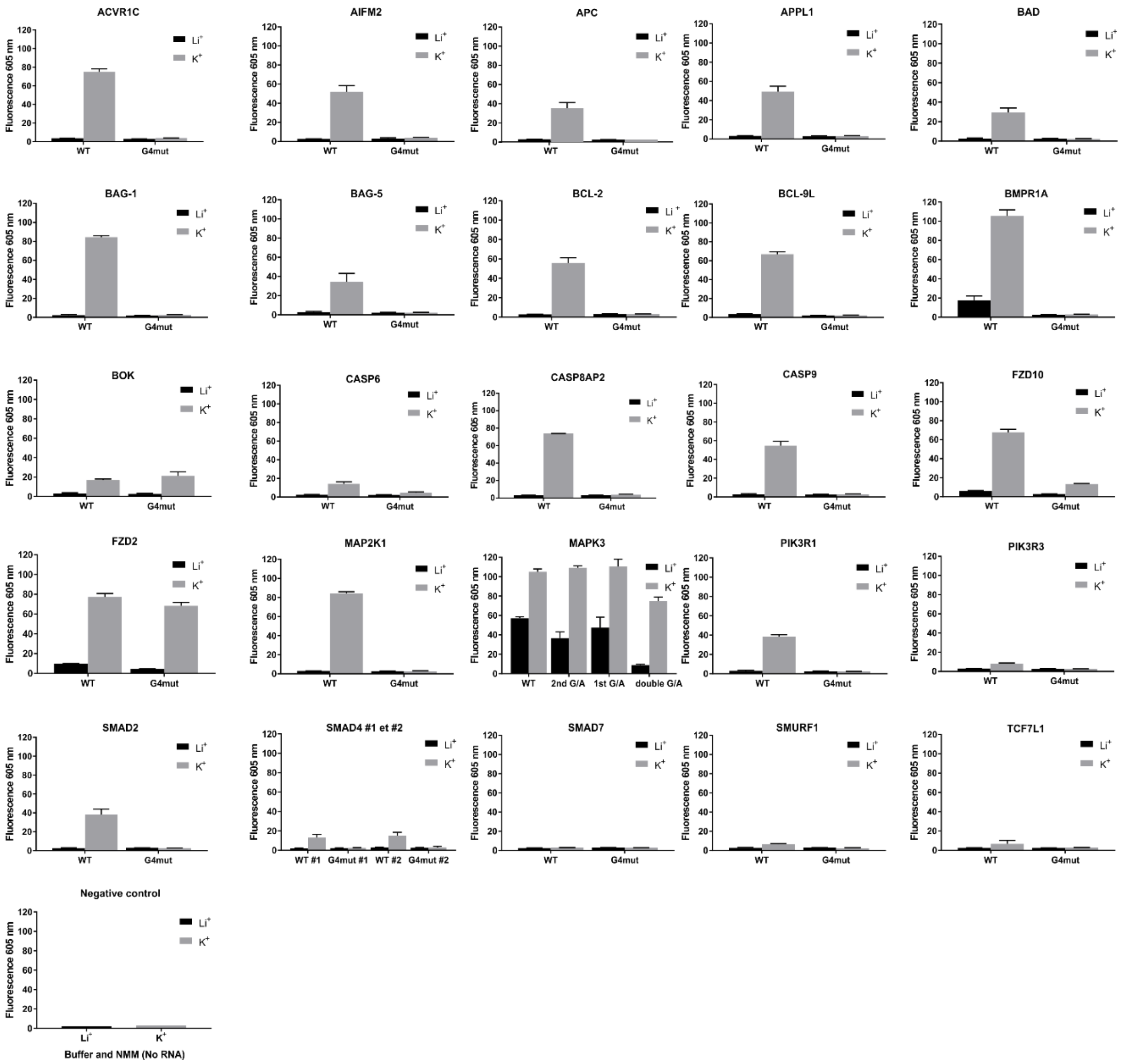

Figure S2 NMM assay of all candidates.

The fluorescence emission peaks at 605 nm under the different conditions: Black Li^+, Gray K^+. Each bar represents the mean of 3 independent experiments and the error bars represent the standard deviations.
