# Supplementary material for: G-quadruplexes formation in the 5’UTRs of mRNAs associated with colorectal cancer pathways
Source: PLoS One. 2018 Dec 3;13(12):e0208363. doi: 10.1371/journal.pone.0208363 (PMC6277105; doi:10.1371/journal.pone.0208363)
Supplement: S3 Fig — Results for A) BAG-1 and B) CASP8AP2 from the Apoptosis set; and C) MAPK3 from the PI3-K set. The results are shown as the means of the Rluc expression normalized over the Fluc transfection control. The WT results are in black and the G/A‑mutants are in different shades of gray. The error bars represent the standard deviations. Statistical difference was measured using an unpaired Student t-test with a n = 2 for APC, n = 3 for BAG-1 and CASP8AP2 and n = 5 for MAPK3. *P-value < 0.05 **P-value < 0.01 ***P-value < 0.001. (PDF) [file pone.0208363.s003.pdf]

Figure S3 (Jodoin & Perreault 2018)

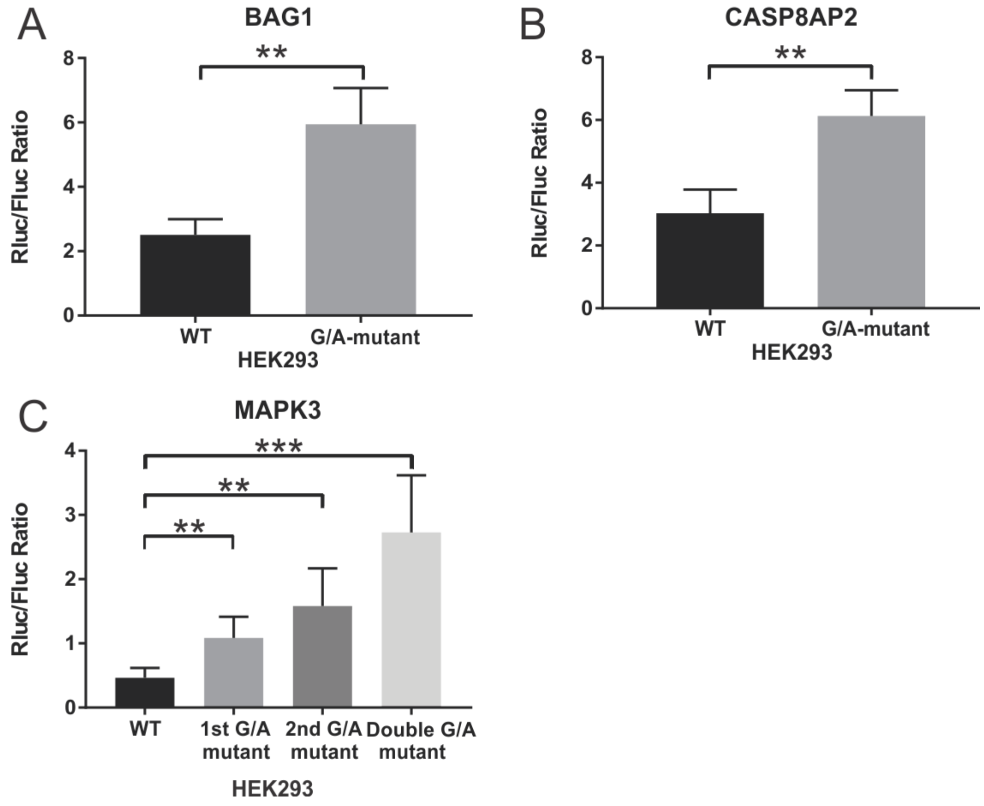

Figure S3 *In cellulo* luciferase assay in HEK293 cells.

Results for A) BAG-1 and B) CASP8AP2 from the Apoptosis set; and, C) MAPK3 from the PI3-K set. The results are shown as the means of the Rluc expression normalized over the Fluc transfection control. The WT results are in black and the G/A-mutants are in different shades of gray. The error bars represent the standard deviations. Statistical difference was measured using an unpaired Student t-test with a n=2 for APC, n=3 for BAG-1 and CASP8AP2 and n=5 for MAPK3. *P-value < 0.05 **P-value < 0.01 ***P-value < 0.001
