# Supplementary material for: G-quadruplexes formation in the 5’UTRs of mRNAs associated with colorectal cancer pathways
Source: PLoS One. 2018 Dec 3;13(12):e0208363. doi: 10.1371/journal.pone.0208363 (PMC6277105; doi:10.1371/journal.pone.0208363)
Supplement: S4 Fig — The WT and the G/A-mutant full-length 5’UTRs were inserted upstream of the Renilla luciferase (Rluc) reporter gene and used for transfection. The G mutated to A were the same as those in the in vitro assays. A) APC, B) BAG-1 and C) CASP8AP2. The results are shown as the means of the Rluc expression normalized over the Fluc transfection control in the three colorectal cell lines HCT116, HT29 and DLD-1. The WT results are in black and the G/A-mutants’ results are in gray. The error bars represent the standard deviations. Statistical difference was measured using an unpaired Student t-test with a n = 3 *P‑value < 0.05 **P-value < 0.01. (PDF) [file pone.0208363.s004.pdf]

Figure S4 (Jodoin & Perreault 2018)

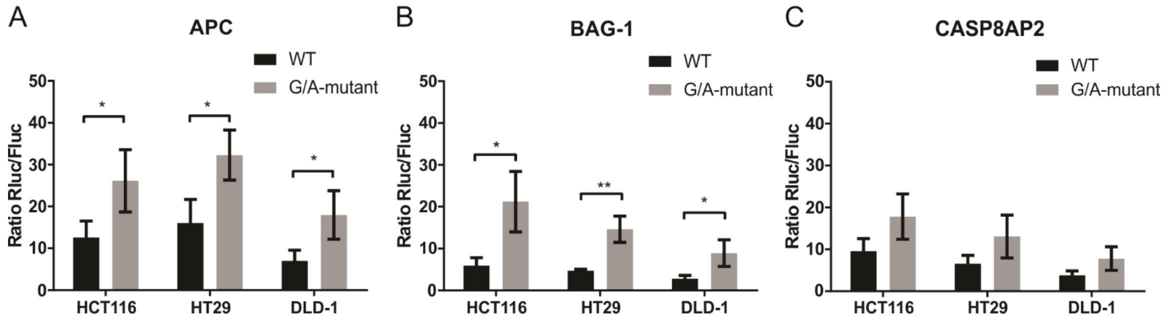

Figure S4 *In cellulo* luciferase assay in colorectal cancer cell lines.

The WT and the G/A-mutant full-length 5’UTRs were inserted upstream of the Renilla luciferase (Rluc) reporter gene and used for transfection. The G mutated to A were the same as those in the *in vitro* assays. A) APC, B) BAG-1 and C) CASP8AP2. The results are shown as the means of the Rluc expression normalized over the Fluc transfection control in the three colorectal cell lines HCT116, HT29 and DLD-1. The WT results are in black and the G/A-mutants’ results are in gray. The error bars represent the standard deviations. Statistical difference was measured using an unpaired Student t-test with a n=3 *P-value < 0.05 **P-value < 0.01
