# Supplementary material for: G-quadruplexes formation in the 5’UTRs of mRNAs associated with colorectal cancer pathways
Source: PLoS One. 2018 Dec 3;13(12):e0208363. doi: 10.1371/journal.pone.0208363 (PMC6277105; doi:10.1371/journal.pone.0208363)
Supplement: S2 Table — (PDF) [file pone.0208363.s006.pdf]

S2 Table Comparison of the prediction methods

Predictions tools	G4 predictions	dsRNA predictions	TP	FP	TN	FN	Sensitivity	Specificity
cG/cC	13	13	10	3	8	5	0.66	0.72
G4H	6	20	5	1	10	10	0.33	0.90
G4NN	10	16	9	1	10	6	0.60	0.90
RNAfold	16	10	10	6	5	5	0.66	0.45
In vitro	+	-						
Confirmation	15	11						

TP: True positive, FP: False positive, TN: True negative, FN: False negative
